# Nanomedicine-based theranostics in atherosclerotic cardiovascular diseases

**DOI:** 10.1186/s12929-026-01245-y

**Published:** 2026-05-02

**Authors:** Chih-Fan Yeh, Laura Ianalieva, Hong-Kong Wong, Chau-Chung Wu, Kai-Chien Yang

**Affiliations:** 1 Division of Cardiology, Department of Internal Medicine and Cardiovascular Center, National Taiwan University Hospital, Taipei, Taiwan; 2 Institute of Biological Chemistry, Academia Sinica, Taipei, Taiwan; 3 Department and Graduate Institute of Pharmacology, National Taiwan University College of Medicine, No.1, Sec. 1, Ren-Ai Rd, 1150R Taipei, Taiwan; 4 Department & Graduate Institute of Medical Education & Bioethics, College of Medicine, National Taiwan University, Taipei, Taiwan; 5 Department of Internal Medicine, Yonghe Cardinal Tien Hospital, New Taipei City, Taiwan; 6 Research Center for Developmental Biology & Regenerative Medicine, National Taiwan University, Taipei, Taiwan; 7 Center for Frontier Medicine, National Taiwan University Hospital, Taipei, Taiwan; 8 Institute of Biomedical Sciences, Academia Sinica, Taipei, Taiwan; 9 National Taiwan University Advanced Biomedical Research Center, Taipei, Taiwan

**Keywords:** Atherosclerosis, Nanomedicine, Nanoparticle, Theranostics

## Abstract

Current treatment for atherosclerotic cardiovascular diseases (ASCVD) mainly focuses on the modification of systemic risk factors, such as hyperglycemia and hyperlipidemia. Despite significant efforts and expanse, achieving early and proper diagnosis of ASCVD to improve clinical outcomes remains challenging, and vascular-targeted therapies or genetic editing, while ideal, are still limited. The development of nanomedicine-based mRNA vaccines for SARS-CoV-2 has demonstrated the potential of nanotechnology to target previously inaccessible molecules. Precision therapies by nanomedicine targeting specific tissues/molecules hold potential for new treatment paradigms by precisely modulating disease-causing molecular pathways within diseased tissues, including dysfunctional vasculature. By leveraging insights into the pathogenic contributors of atherogenesis, researchers have optimized nanoplatforms’ composition, synthesis strategies, and surface design to enhance therapeutic efficacy and enable early diagnosis. Herein, we present an updated overview of therapeutic and diagnostic strategies using nanomedicine for ASCVD, and explore future research directions and innovative approaches for nanomedicine-driven theranostics in cardiovascular care.

## Background

Atherosclerotic cardiovascular disease (ASCVD) is a major contributor to cardiovascular morbidity and mortality and remains the leading cause of death globally [1, 2]. It is a systemic, chronic, and progressive condition characterized by lipid accumulation, persistent inflammation, and vascular remodeling. These pathological processes contribute to the development of atherosclerotic plaques, which can lead to a range of vascular complications such as myocardial infarction, stroke, and peripheral artery disease [3–5]. Treatment strategies for ASCVD are multifaceted, aiming to reduce modifiable risk factors, slow disease progression, and prevent recurrent cardiovascular events. Preventive lifestyle interventions, including adherence to a heart-healthy diet, regular physical activity, smoking cessation, and weight management, are fundamental in mitigating systemic risk factors. In parallel, pharmacological and interventional approaches play a critical role, encompassing lipid-lowering therapy, antithrombotic agents, plaque stabilization strategies, and coronary revascularization via percutaneous coronary intervention (PCI) or coronary artery bypass grafting (CABG) [6]. Recent studies have demonstrated that anti-inflammatory therapies can significantly reduce the risk of ischemic cardiovascular (CV) events in patients at high or very high risk, supporting the feasibility of mechanism-targeted therapeutic strategies beyond traditional risk factor management [7–9]. However, these clinical trials have also reported an increased incidence of infections, particularly pneumonia among patients receiving anti-inflammatory treatments, raising important safety concerns regarding immunosuppression in this population. Moreover, despite substantial advances and ongoing investments in ASCVD therapies, a considerable residual risk of adverse CV events persists, highlighting current challenges in the early detection of high-risk patients and the need for novel and safer treatment approaches. Despite advances in our understanding of atherosclerosis biology, progress remains limited. Notably, approximately half of the individuals who suffer sudden cardiac death exhibit no prior symptoms. Key pathophysiological mechanisms underlying acute coronary syndrome (ACS) include plaque rupture, plaque erosion, calcified nodules, and functional alterations in the coronary arteries [10].

In addition, Current clinical imaging techniques primarily focus on assessing plaque morphology, size, and overall composition. However, these modalities provide limited information on the molecular and cellular features of atherosclerotic plaques, which are critically associated with future cardiovascular events. Beyond local hemodynamic forces, recent evidence suggests that the activation of intimal endothelial cells, neutrophils, and the formation of neutrophil extracellular traps, driven by innate immune responses, plays a more prominent role in thrombosis linked to superficial erosion than in plaque rupture. This highlights inflammatory pathways as promising targets for both diagnostic innovation and therapeutic intervention [11, 12]. Therefore, the development of novel imaging technologies and molecular probes could enable earlier diagnosis and provide complementary benefits to existing therapies, ultimately improving the clinical management of atherosclerosis.

Nanotechnology offers distinct advantages in addressing these unmet needs in ASCVD management. Nanomedicine has emerged as a promising platform for precise and targeted theranostics and interventions across the spectrum of ASCVD. Numerous studies have shown that nanoparticles (NPs) can enhance the pharmacokinetic profile and chemical stability of loaded therapeutics while reducing off-target effects and systemic toxicity. In addition, targeted NPs have been engineered to improve the delivery of imaging agents to specific molecular and cellular components within atherosclerotic plaques, thereby enabling more accurate assessment of plaque composition and stability.

To elucidate the role of nanotechnology in ASCVD theranostics, this review will first examine the pathophysiological basis and cellular targets of the disease. It will then explore NP-based therapeutic platforms, including inorganic, organic, biomimetic, and hybrid systems that facilitate diagnostic and therapeutic applications. These platforms target various pathological processes, such as reactive oxygen species (ROS), lipid metabolism, chronic vascular inflammation, and macrophages and smooth muscle cells, which will be explored. Finally, we summarize the current clinical trial landscape of nanomedicine and discuss the remaining challenges and future directions for its integration into ASCVD management.

## Pathophysiology of ASCVD and potential therapeutic targets

**Fig. 1:**
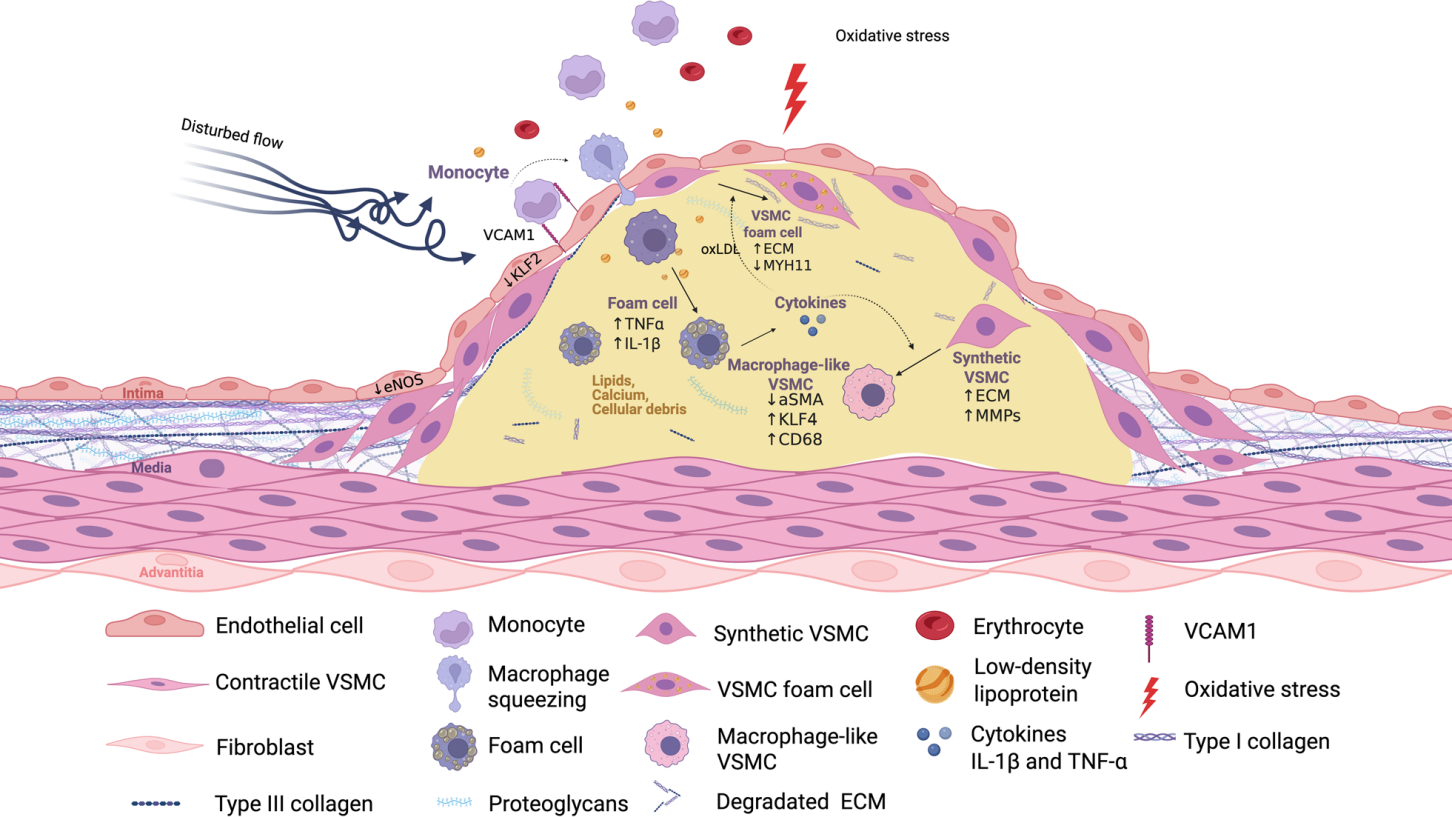
Multicellular pathways that underpin plaque growth and failure. Pathologic stimuli such as disturbed flow (blue arrows) suppress the Krüppel-like factor and endothelial nitric oxide synthase (KLF2-eNOS) axis in the overlying endothelium, induce local oxidative stress (red lightning bolt), and upregulate the expression of vascular cell-adhesion molecule-1 (VCAM-1; purple bars). These changes facilitate the adhesion of circulating monocytes, which infiltrate along the endothelial surface85, transmigrate through widened junctions, and differentiate into macrophages within the intima. There, macrophages encounter and ingest retained lipoprotein particles, oxidize LDL particles (gold), and transform into lipid-laden foam cells. In turn, they release proinflammatory cytokines such as IL-1β and TNF-α, amplifying local inflammatory responses. This proinflammatory medium drives vascular smooth muscle cells (VSMCs; pink spindles) to migrate into the intima and transition into either a synthetic phenotype (rounded pink) or a macrophage-like state (pale pink). Both VSMC variants contribute to a provisional extracellular matrix (blue mesh), and some accumulate lipids to form VSMC-derived foam cells. Over time, collagen cross-linking at the lesion shoulder narrows the lumen, while excessive matrix degradation weakens the fibrous cap, rendering the plaque prone to rupture or surface erosion. Together, these interconnected endothelial, lipid, immune, macrophage, and VSMC pathways represent critical targets for emerging nanotherapeutic strategies aimed at modulating atherosclerotic disease progression

Atherosclerosis is a complex, multifactorial, and progressive disease driven by a dynamic interplay of metabolic, inflammatory, and biomechanical processes within the arterial wall (Fig. 1). Disturbed hemodynamics, metabolic overload, and aging-associated oxidative stress affect the endothelium, suppressing the Krüppel-like factor and endothelial nitric oxide synthase (KLF2-eNOS) axis. This suppression reduces local nitric oxide (NO) bioavailability and triggers a dramatic increase in reactive oxygen species (ROS) production [13–15]. The resulting oxidative environment upregulates vascular cell adhesion protein 1 (VCAM-1), disrupts endothelial junctional integrity, and enables the recruitment and transendothelial migration of circulating monocytes and lymphocytes into the intima [16]. Within this lipid-rich niche, retained and progressively oxidized low-density lipoproteins (oxLDLs) drive monocyte polarization into proinflammatory macrophages. Impaired efferocytosis then leads to the conversion of these cells into foam cells, which amplify local inflammation through the secretion of cytokines such as IL-1β and TNF-α, as well as matrix-degrading proteases [17]. Concomitantly, vascular smooth muscle cells (VSMCs) undergo a phenotypic switch, losing their contractile phenotype, migrating into the intima, and adopting synthetic or even macrophage-like identities. These altered VSMCs contribute to extracellular matrix (ECM) deposition and, under sustained inflammatory signaling, may participate in microcalcification [18]. Initially, outward (positive) vascular remodeling compensates for lesion growth. However, progressive collagen-rich ECM accumulation and an imbalance favoring matrix metalloproteinases (MMPs) over their inhibitors lead to ECM degradation, fibrous cap thinning, and increasing plaque vulnerability [19–21]. Disruption of the fibrous cap exposes the thrombogenic lipid core, precipitating acute coronary syndromes and ischemic stroke.

Each node in this self-reinforcing cascade can also be viewed as a therapeutic target: reactivating endothelial mechanosensing, enforcing cholesterol efflux, tempering IL-1β-driven inflammation, reinstating a contractile VSMC phenotype, or rebalancing ECM turnover. Such precision demands delivery systems that release cargo only where and when it is needed. Stimuli-responsive NPs engineered to recognize lesional acidosis, excess ROS, or cell-specific receptors enable simultaneous endothelial repair, immune reprogramming, and matrix stabilization. The following sections survey inorganic, organic, and biomimetic nanomaterials that exploit these pathophysiological cues to diagnose and treat atherosclerotic cardiovascular diseases.

## Literature search strategy

A comprehensive literature search was conducted across PubMed and Google Scholar for studies published between 2010 and 2025. The search strategy employed combinations of keywords, including nanoparticles, atherosclerosis, and the composition and synthesis of nanoparticle platforms. Keywords related to nanomedicine therapeutics for ASCVD included nanoparticles, macrophage-based systems, lipid-based platforms, monocyte-based delivery systems, ROS-sensitive, pH-sensitive, mechanosensing, mechanosensitive, mechanotransduction, flow-dependent signaling, and hemodynamics. The keywords “nanoparticles,” “atherosclerosis,” and “diagnostic imaging” were used to identify studies on nanoparticle-based diagnostic imaging. Additional keywords included nanoparticles, cholesterol, clinical trials, atherosclerosis, and atherosclerotic plaque to identify clinical studies on nanomedicine-based therapies. This review focuses on the biophysicochemical properties of therapeutic platforms and the mechanisms of active and passive targeting for diagnostic imaging strategies in ASCVD. Studies were excluded if they lacked detailed NP characterization or focused on non-cardiovascular pathologies, to ensure a focused scope on nanomedicine-based theranostics in atherosclerosis. All keywords were first searched in the title and abstract fields and subsequently across all database fields.

## NP-based therapeutic platforms for ASCVD: composition, synthesis, and biophysicochemical properties

At a mechanistic level, plaque-seeking NPs are trilayered constructs: a core that governs size and stiffness, a surface cover that determines pharmacokinetics and guides the particle toward the inflamed endothelium, and an encapsulated or conjugated cargo that is released only upon encountering a plaque-specific trigger. In the following sections, we classify these systems based on the nature of the core, such as inorganic, organic, biomimetic, or hybrid, because this structural element ultimately dictates the suite of chemistries, stimuli-responsiveness, and manufacturing pathways available for ASCVD theranostics (Table 1).

**Table 1:** Description of different nanoparticles used in ASCVD

Type of nanomaterial	Type of NPs		Size	Description	Properties	References
Inorganic Nanomaterials	Metallic NPs	Iron oxide-based NPs (Fe_3_O_4_)	10–20 nm	Due to the properties and a large surface area, IONPs are widely used in drug delivery, bioengineering, and imaging (esp. MRI)	Magnetism, good biocompatibility, and versatility	[27, 28]
		Cerium oxide-based NPs	20–30 nm	Cerium oxide exists in both + 3 and + 4 states, which form CeO_2_ (CeO_2_ NPs)	Antioxidant anti-inflammatory	[5, 31]
		Gold-base NPs	1–100 nm	It is usually used in hybrid systems integrated with liposomes and polymers NPs, or surface modifications in biomedical applications	Good biocompatibility, targeting precision, drug-loading, and low cytotoxicity	[33, 34]
	Non-metallic NPs	mesoporous silica-based NPs	10–600 nm	It is featured with a substantial surface area, a unique mesh-like pore structure, and regular pore channels that can be adjusted to meet specific needs as nanocarriers	Good biocompatibility, optimizing effective and safe drug delivery systems	[1, 38]
		Carbon-based NPs	1–100 nm	A wide range of innovative carbon-based nanomaterials is used for graphene, carbon nanotubes, nanodiamonds, and nanodots	High photothermal conversion FL effects, high drug loading, and low cytotoxicity	[41, 43]
Organic Nanomaterials	Lipid-Based NPs	Liposomes	50–500 nm	It is featured with a lipophilic bilayer sandwiched between two hydrophilic layers, which enables to encapsulate hydrophobic molecules within the lipid bilayer while enclosing hydrophilic agents in the central aqueous core	Preventing drug degradation, improving solubility and bioavailability of drugs, good biodistribution, and delivering drug targeting	[48, 49]
		HDL-like NPs	7–13 nm	HDL primarily exists in two structural forms such as d-HDL and s-HDL. d-HDL has a phospholipid bilayer, while s-HDL features a phospholipid monolayer with a hydrophobic core	Low toxicity, good drug delivery, and good biocompatibility	[53–55]
		LDL-like NPs	18–25 nm	It consists of a hydrophilic phospholipid mono-outer layer with apoB-100 and free cholesterol, as well as an inner hydrophobic core with esterified cholesterol and triacylglycerol	Good biosafety, biocompatibility, and biodegradability	[58, 59]
	Polymeric NPs	Natural polymers (Dextran, cyclodextrins, chitosan)	1–1000 nm	It is mainly consisted of algae, plant, microorganisms (dextran), and animal (chitosan) products	Good biocompatibility, biodegradability, and targeting delivery	[62, 63, 65]
		Synthetic polymers (PLGA)		It is characterized by a high specific surface area and quantum size effects, making it suitable for use as a drug delivery carrier and in tissue engineering	Good biocompatibility, biodegradability, improving the hydrophobicity, efficacy, and toxicity of drugs	[68, 69, 71]
		PEG-NPs	~ 50 nm	Surface modification of NPs with PEG is a strategy used to improve therapeutic efficacy and enhance the targeted delivery of drugs	Good biocompatibility, low toxicity, improving the efficiency, targeting delivery, and decreasing adverse effect of drugs	[73, 74]
		Polymer micelles	10–100 nm	It is characterized by a hydrophobic core and a hydrophilic coating, which allows drugs to be inside the NPs	High encapsulation efficiency, easy preparation, prolonged drug retention, and good biocompatibility	[75, 77]
Biomimetic Nanomaterials	Cell membrane-based NPs	Erythrocyte membrane-coated NPs	70–200 nm	It can be encapsulated by drugs or other compounds as carriers	Long-term circulation, low toxicity, good biocompatibility and biodegradability, and long lifespan	[80, 81]
		Platelet membrane-coated NPs	~ 130 nm	It combines platelet functions for targeted drug delivery	Immune escape and good targeting capability	[85, 86]
		Macrophage membrane-coated NPs	~ 90 nm	It consisted of a thin layer of macrophage membrane-coating NPs as the core and the cell membrane as the outer shell	Good targeting delivery to the atherosclerotic lesion site and a cleaner for proinflammatory factors	[90–92]
		Monocytes membrane-coated NPs	< 200 nm	Drug or therapeutic compounds coated with monocyte membrane NPs can specifically target atherosclerotic sites, thereby enhancing cellular targeting, receptor binding, and intracellular uptake	Enhancing targeting cell, intracellular uptake, and specifically binding cell receptor in the stage of diseases	[94, 95]

NPs: nanoparticles; Fe_3_O_4_: ferri-ferrous oxide; IONPs: Iron oxide-based NPs; MRI: magnetic resonance imaging; FL: fluorescence; HDL: high-density lipoprotein; d-HDL: discoidal HDL; s-HDL: spherical HDL; LDL: low-density lipoprotein; apoB: apolipoprotein B; PEG: polyethylene glycol

### Overview of inorganic NPs

Inorganic nanoparticles (NPs)—including gold, iron oxide, and silica-based platforms—possess unique physicochemical properties such as superparamagnetism for MRI-based molecular imaging, photoresponsiveness for photothermal therapy, and inherent catalytic activity that enables them to act as nanozymes. Beyond serving as stable structural frameworks, these platforms offer high therapeutic relevance in cardiovascular medicine by enabling site-specific delivery that overcomes the limitations of systemic drug administration, such as off-target toxicity and rapid clearance. Mechanistically, these NPs utilize surface-anchored ligands (e.g., peptides or antibodies) to achieve high-affinity binding with biomarkers like VCAM-1 or αvβ3 integrins, which are overexpressed on activated endothelial cells and macrophages within atherosclerotic plaques. Once localized at the lesion, these NPs provide cardiovascular-specific benefits by modulating the local microenvironment; they can be engineered to suppress pro-inflammatory NF-κB signaling, neutralize reactive oxygen species (ROS), or upregulate ABCA1-mediated cholesterol efflux pathways, thereby promoting plaque stabilization and reducing the risk of acute myocardial infarction [22]. Compared with organic alternatives, inorganic NPs are also easier to synthesize and manufacture consistently, making them well-suited for biomedical applications, especially in the treatment of ASCVD [23]. Within the landscape of ASCVD theranostics, these materials are broadly categorized into *metallic NPs*, such as Iron oxide-based NPs (IONPs) for contrast-enhanced imaging, cerium oxide for ROS scavenging, and gold-based NPs for photothermal applications, as well as *nonmetallic NPs*, including mesoporous silica and carbon-based NPs, which offer high surface areas for maximized drug loading.

### Metallic NPs

Metal and metal-derivative NPs —most notably those based on gold, iron, and cerium—exhibit high surface-area-to-volume ratios and unique catalytic and immunomodulatory properties. While these materials are established in cell sorting and diagnostic magnetic resonance imaging (MRI) [24, 25], their therapeutic relevance in cardiovascular medicine has expanded toward active lesion modulation. These systems have shown significant potential in targeting atherosclerotic plaques, enhancing thrombolysis, and improving cardiac repair post-myocardial infarction. Additionally, metallic NPs are utilized for targeted drug delivery owing to their magnetic responsiveness and superparamagnetic behavior [26].

#### Iron oxide-based NPs (IONPs)

IONPs, particularly magnetite (Fe3O4), are distinguished by their superparamagnetism, biocompatibility, and high surface-to-volume ratio. Typically synthesized in the 10–20 nm range [27], these particles exhibit high magnetic susceptibility, allowing for precision localization within the vasculature when guided by external magnetic fields—a feature with significant therapeutic relevance for concentrated drug delivery to high-shear arterial regions [24, 29]. Fe_3_O_4_ can be synthesized through various chemical and physical methods, including coprecipitation, sol–gel synthesis, thermal decomposition, vapor/aerosol-phase techniques, and pulsed laser deposition [27]. While IONPs are gold-standard contrast agents for T2-weighted MRI [28], uncoated (or 'naked') IONPs often exhibit dose-dependent cytotoxicity due to the generation of ROS via the Fenton reaction [29]. To mitigate this, surface modification with biocompatible materials is essential for reducing cytotoxicity and enhancing physiological compatibility. For example, biomimetic Fe_3_O_4_ NPs coated with macrophage membranes (Fe_3_O_4_ @M) have been developed for imaging applications aimed at detecting early atherosclerotic lesions, while uncoated IONPs can exhibit cumulative toxicity. This targeting mechanism relies on the VCAM-1/integrin system, where membrane-bound α4β1 integrins facilitate molecular recognition and recruitment of the NPs into the activated endothelium [24]. These Fe_3_O_4_ @M NPs exhibit improved biosafety and effectively target early-stage atherosclerotic lesions via integrin α4β1-mediated recognition of VCAM-1 [30]. Thus, integrating iron oxide NPs with surface modification strategies holds significant promise for advancing diagnostic and therapeutic approaches in atherosclerosis.

#### Cerium oxide-based NPs

Cerium oxide-based (CeO_2_) NPs have have emerged as potent nanozymes for ASCVD therapy due to their inherent antioxidant and anti-inflammatory capacities [5]. Typically 20 to 30 nm in size, CeO_2_ NPs possess a unique surface chemistry characterized by the coexistence of Ce^3+^ and Ce^4+^ oxidation states. Mechanistically, cerium oxide-based NPs function through a self-regenerative catalytic cycle; owing to a low reduction potential, the NPs switch between Ce^3+^ and Ce^4+^ states to neutralize superoxide radicals and hydrogen peroxide [31], effectively mimicking the activity of endogenous superoxide dismutase (SOD) and catalase. These NPs can be synthesized through a variety of physical and chemical methods, including precipitation/coprecipitation, sonochemistry, and the sol–gel method [32]. Hyaluronic acid (HA)-encapsulated cerium dioxide (HA-CeO_2_) NPs are specifically engineered to scavenge ROS via both superoxide dismutase (SOD)-mimetic and catalase-mimetic activities. Furthermore, by transiently decreasing molecular oxygen levels, these NPs can induce HIF1α stabilization to regulate genes associated with angiogenesis and tissue repair [31]. Studies have demonstrated that HA-CeO_2_ NPs exhibit robust SOD-mimetic function, significantly reducing ROS levels, as confirmed by water-soluble tetrazolium 8 (WST-8) and ROS scavenging assays. Notably, these NPs also display self-regenerating antioxidant activity, resulting in a reduction of aortic plaque area by approximately two-thirds and a significant decrease in low-density lipoprotein cholesterol (LDL-C) levels through the disruption of lipid metabolism [5]. In summary, cerium dioxide NPs represent a promising therapeutic platform that can synergize with conventional pharmacological agents like statins to reduce the plaque burden in ASCVD.

#### Gold-based NPs (AuNPs)

AuNPs have shown great potential in the management of atherosclerosis because of their tunable composition, diverse synthesis methods, and unique biophysicochemical interactions. The mechanism of action is largely governed by surface plasmon resonance, where the fluctuation of surface electrons allows the particles to absorb near-infrared light and convert it into localized thermal energy. This property enables photothermal therapy, which can be leveraged to selectively induce apoptosis in inflammatory macrophages or "melt" lipid-rich cores within vulnerable plaques. Structurally, AuNPs, ranging in size from 1 to 100 nm, can adopt various shapes. Furthermore, AuNPs are used in hybrid systems integrated with liposomes or polymers to enhance functionality [33, 34]. Surface modifications play a critical role in their biomedical applications, with ligands such as polyethylene glycol (PEG), peptides, or oligonucleotides improving biocompatibility, targeting precision, and drug-loading capacity. At the cellular level, these NPs reduce endothelial dysfunction by downregulating endothelin-1 vasoconstrictor and suppressing the proliferation of VSMC through mitochondrial activity modulation. These surface adaptations also enable diagnostic-therapeutic applications by facilitating interactions with biological components while minimizing cytotoxicity at therapeutic doses [35]. For instance, silica-gold NPs have achieved over a 40% reduction in atherosclerotic lesions, whereas traditional statin therapies or stent placements often result in reductions of less than 13% [25]. Naked AuNPs (0.5–50 nm) can be synthesized via physical methods (pAuNPs) and have been shown to reduce endothelial dysfunction by downregulating the vasoconstrictor endothelin-1, suppressing VSMC proliferation via the modulation of mitochondrial activity, and inhibiting inflammatory responses in plaque macrophages [36].

### Nonmetallic NPs

Nonmetallic NPs, primarily composed of nonmetallic elements such as mesoporous silica and carbon-based materials, are characterized by low toxicity, high biocompatibility, and efficient drug delivery performance. These nanomaterials have emerged as pivotal tools in managing ASCVD by providing precise targeting to inflammatory macrophages and foam cells that drive plaque progression. Nonmetallic NPs enable stimuli-responsive drug release triggered by the unique microenvironment of atherosclerotic plaques. By integration of advanced imaging with therapy, these NPs facilitate a theranostic approach that allows for real-time monitoring of treatment efficacy and plaque vulnerability, ultimately improving clinical outcomes in vascular disorders.

#### Mesoporous silica-based NPs (MSNs)

MSNs are advantageous as effective and safe drug delivery systems because of their high biocompatibility, large pore surface area, small particle size, and high drug-loading capacity [1]. Typically, ranging from 10 to 600 nm in particle size and 2 to 50 nm in pore size, MSNs can be synthesized through various methods, including sol–gel, microwave-assisted, hydrothermal, and modified aerogel techniques [37]. MSNs feature a substantial surface area, a unique mesh-like pore structure, and uniform pore channels that can be tailored to meet specific therapeutic requirements. This versatility enhances their effectiveness as nanocarriers, improving drug water solubility and minimizing side effects [38]. A notable example is IL-1Ra@Cu-MSNs, which co-deliver copper ions (Cu^2^⁺) and an interleukin-1 receptor antagonist (IL-1Ra) to mitigate copper-induced hepatic damage. Both in vitro and in vivo studies demonstrate that IL-1Ra@Cu-MSNs significantly reduce inflammatory responses, the plaque area, and macrophage infiltration compared to Cu-MSNs and IL-1Ra alone, highlighting the superior therapeutic efficacy of MSNs [39]. In summary, the unique functional and structural properties of MSNs make them highly promising candidates for future atherosclerosis treatment strategies.

#### Carbon-based NPs

Carbon-based NPs are available in various shapes and forms, typically with sizes ranging from 1 to 100 nm. In recent years, a wide range of innovative carbon-based NPs—such as graphene, carbon nanotubes, nanodiamonds, and carbon nanodots—have attracted significant attention due to their unique structural and functional properties [40]. These properties include high photothermal conversion efficiency and distinct fluorescence (FL) characteristics, making them suitable for applications such as photothermal therapy (PTT), photoacoustic imaging (PAI), and fluorescence tracing [41]. Common synthesis methods for carbon-based nanomaterials include electric arc discharge, photoablation, and chemical vapor deposition [42]. Among these materials, carbon nanocages are particularly noteworthy because of their high drug-loading capacity and low cytotoxicity [43]. Chitosan carbon nanocages (CS-CNCs@Ce6/DS), modified with dextran sulfate (DS) to deliver chlorin e6 (Ce6), have demonstrated substantial drug-carrying capacity. In in vivo experiments in which *ApoE*⁻/⁻ mice were subjected to PTT, the CS-CNC@Ce6/DS group exhibited a substantial reduction in the plaque area via ablation of the activated macrophages [44]. In summary, carbon-based NPs can reduce drug side effects by enabling lower drug dosages through their high drug-loading capacity.

### Organic nanomaterials

, organic NPs, such as liposomes, high-density lipoprotein (HDL)-like NPs, LDL-like NPs, polymeric NPs, and PEG-NPs, offer several advantages. These include high drug-loading capacity, low toxicity, excellent biocompatibility, prolonged circulation time in the bloodstream, and rapid elimination from the body, making them well-suited for drug delivery applications [45]. Furthermore, their capacity for active targeting allows for the selective delivery of therapeutic agents, genetic materials, and imaging contrast agents, which is crucial for stabilizing plaques and reducing disease burden. However, organic NPs also present certain limitations, including high production costs, long-term safety concerns, stability challenges, and nonspecific drug release, all of which limit their clinical applicability [46].

#### Lipid-based NPs

Lipid-based nanoparticles (LNPs) are promising therapeutic tools for the treatment of ASCVD. Owing to their structural and physicochemical properties, LNPs are well-suited for encapsulating drugs or bioactive compounds used in ASCVD therapy. Lipid-based NPs function by creating a biocompatible shield for drugs, which prevents premature metabolic degradation and leverages their lipophilic nature to enhance integration with target cell membranes. Furthermore, LNPs can protect drugs from degradation and increase their bioavailability [47]. Common types of LNPs include liposomes, HDL-like NPs, and LDL-like NPs.

#### Liposomes

Liposomes, which range in size from 50 to 500 nm, are lipid-based spherical vesicles characterized by a lipophilic bilayer sandwiched between two hydrophilic layers. This unique architecture enables them to encapsulate hydrophobic molecules within the lipid bilayer and hydrophilic agents within the central aqueous core, effectively protecting these substances from degradation [48]. Various methods are employed in liposome synthesis, each producing vesicles of distinct sizes, degrees of lamellarity, and vesicularity tailored to specific applications. These methods include thin-film hydration, ethanol injection, reverse-phase evaporation, and detergent depletion [49]. In addition to protecting drugs from degradation, liposomal NPs enhance drug delivery in several ways, such as improving drug solubility and bioavailability, increasing biodistribution, and enabling better targeting [50]. Liposomes are widely used in gene delivery therapies for atherosclerosis. The primary mechanism of action for liposomes involves direct fusion with target cells or receptor-mediated uptake, ensuring the intracellular release of payloads that effectively reduce the secretion of inflammatory cytokines and mitigate foam cell formation. For example, PEGylated cationic liposomes delivering miR-146a, a microRNA known to suppress inflammatory responses, through inhibition of intercellular adhesion molecule 1 (ICAM-1) expression and decreased monocyte adhesion. Moreover, this type of NP significantly enhanced the stability and transfection efficiency of miR-146a. Consequently, targeting moieties on liposomes can increase their accumulation in atherosclerotic plaques, thereby increasing their therapeutic efficacy [51]. In conclusion, the incorporation of targeting peptides into liposomes significantly improves their ability to deliver drugs to various cell types within plaques, thereby improving therapeutic outcomes.

#### HDL-like NPs

HDL-like NPs, with a small size range of 7–13 nm, have numerous applications in atherosclerosis treatment [52]. HDL primarily exists in two structural forms in the body: discoidal HDL (d-HDL) and spherical HDL (s-HDL). d-HDL possesses a phospholipid bilayer, whereas s-HDL features a monophospholipid layer surrounding a hydrophobic core. d-HDL is mainly secreted by the liver, where it facilitates cholesterol uptake and interacts with plasma lecithin-cholesterol acyltransferase (LCAT). This interaction converts d-HDL into s-HDL, which is then transported to the liver for excretion [53]. Compared with native HDL, recombinant HDL (rHDL), a synthetic form of HDL, is less toxic, more stable, and easier to synthesize. However, the two types of rHDL-like NPs—s-rHDL and discoidal d-rHDL—differ in their biophysicochemical properties: (1) s-rHDL is more stable than d-rHDL; (2) s-rHDL contains a distinct hydrophobic core that creates a water-resistant environment; (3) d-rHDL is easier to synthesize; and (4) d-rHDL is more structurally amenable to conjugation with other materials [54]. Furthermore, s-rHDL is more effective than d-rHDL for drug delivery [55]. In a recent study, s-rHDL was engineered to deliver exogenous ganglioside GM3 membrane-bound glycosphingolipid. The GM3—s-rHDL nanoplatform demonstrates superior therapeutic potency by exploiting the selective binding of ApoA-I to macrophage receptors, ensuring precise delivery of the ganglioside payload directly to established lesions. Notably, the therapeutic efficacy observed in the low-dose GM3—s-rHDL group was comparable to that of the high-dose free GM3 group, indicating that s-rHDL enhances GM3 efficacy while reducing its potential side effects through dose minimization [56]. In contrast, d-rHDL has been combined with hyaluronic acid–ferrocene (HA-FC) to create a NP platform for simvastatin delivery (HA-FC/NP3ST). In vivo results revealed that HA-FC/NP3ST exhibited greater therapeutic efficacy in treating atherosclerosis. This targeted interaction with lesional macrophages amplifies cholesterol efflux pathways, offering a significant advantage over non-targeted statin therapies compared to the control [57]. These findings highlight the significant potential of d-rHDL when it is integrated with complementary materials.

#### LDL-like NPs

LDL is a spherical molecule with a diameter of 18–25 nm that is composed of a hydrophilic phospholipid mono-layer containing apolipoprotein B-100 (apoB-100) and free cholesterol and an inner hydrophobic core containing esterified cholesterol and triacylglycerol [58]. Drugs can be incorporated into various structural components of LDL, including the hydrophilic phospholipid layer, hydrophobic core, or apoB-100 [59]. LDL-like NPs, also referred to as aposomes, are engineered with apoB-100 on their surface and are typically synthesized via thin-layer evaporation/extrusion (TLE) or microfluidic methods. These NPs rely on LDL receptor-mediated endocytosis for drug release from lysosomes. Furthermore, LDL-like NPs can achieve targeted drug delivery while avoiding activation of the immune system. They exhibit favorable properties such as high biosafety, biocompatibility, and biodegradability [60]. In a previous study, paclitaxel-loaded LDL-like NPs (LDE-paclitaxel).

### Polymeric NPs

Polymeric NPs range in size from 1 to 1000 nm and consist of various polymeric components. These NPs present significant opportunities for advancing cardiovascular outcomes due to their physical properties and chemical compositions. These NPs can avoid kidney clearance, demonstrate relatively long circulation times, and allow for the active targeting of atherosclerotic lesions. Drugs or other compounds can either be adsorbed onto their surface or encapsulated within their core. Moreover, polymeric NPs can be engineered with customized sizes, shapes, and surface charges [61]. These properties enable them to carry diverse types of cargo, including hydrophobic and hydrophilic compounds, biological molecules, small molecules, proteins, and vaccines. Polymeric NPs are typically classified into four main categories: natural polymers (e.g., dextran, cyclodextrin, and chitosan), synthetic polymers [e.g., poly(lactic-co-glycolic acid) (PLGA)], PEG-based NPs, and polymeric micelles [62].

#### Natural polymers

Natural polymers are derived primarily from algae, plants, microorganisms (e.g., dextran), and animals (e.g., chitosan), and they exhibit superior biocompatibility and biodegradability [63].In addition, they can bind negatively charged drugs through electrostatic interactions for targeted delivery and play a critical role in the treatment of cardiovascular diseases [62]. For instance, they exert anti-atherosclerotic effects by regulating cholesterol metabolism and modulating inflammatory signaling pathways such as NF-kB and MAPK signals, to reduce the macrophage conversion into foam cells. Dextran, a polysaccharide composed of glucose units primarily linked by α-(1 → 6) bonds, is produced by specific bacterial species, such as *Leuconostoc mesenteroides,* through enzymatic processes [64]. It has been investigated for its anti-inflammatory and lipid-lowering effects, which are essential in managing the chronic inflammation and dyslipidemia associated with atherosclerosis. Cyclodextrins, cyclic oligosaccharides derived from starch via enzymatic conversion, possess a hydrophilic outer surface and a hydrophobic cavity, enabling them to form inclusion complexes with various molecules. This feature enhances the solubility and bioavailability of therapeutic agents [65]. Cyclodextrin-based polymers have demonstrated effectiveness in lowering cholesterol levels by promoting cholesterol efflux and inhibiting its absorption, thereby reducing atherosclerotic risk factors [66]. Chitosan, a natural polymer obtained through the deacetylation of chitin found in crustacean exoskeletons, has unique physicochemical properties that facilitate interactions with negatively charged molecules such as lipids and inflammatory mediators. Furthermore, chitosan-cyclodextrin complexes have shown enhanced antimicrobial properties and biofilm inhibition, potentially supporting vascular health by preventing secondary infections in atherosclerotic lesions [67]. Natural polymers demonstrate significant bioactivity when functionalized or combined with NPs.

#### Synthetic polymers (PLGA)

Synthetic polymeric NPs are synthesized through chemical or physical methods and can be classified as either biodegradable or nonbiodegradable [68]. Owing to their nanoscale dimensions and unique physicochemical properties, such as excellent biodegradability, high specific surface area, and quantum size effects, synthetic polymeric NPs can improve therapeutic efficacy and reduce adverse effects [69]. PLGA is an FDA-approved, biodegradable, and biocompatible synthetic polymer characterized by a long half-life. PLGA-based NPs have shown potential as drug delivery carriers and in tissue engineering because of their ability to increase drug hydrophobicity, increase therapeutic efficacy, and reduce drug-associated toxicity [70]. Although curcumin and its natural bioenhancer, bioperine, have demonstrated benefits in the treatment of atherosclerosis, both compounds suffer from poor water solubility and limited bioavailability. PLGA NPs co-delivering curcumin and bioperine have been shown to significantly improve the bioavailability of curcumin. Co-delivery of Bioperine can improve the curcumin effects of cholesterol reduction in foam cells [71]. Furthermore, PLGA NPs can enhance drug safety, thereby increasing their clinical applicability. Owing to their bioadhesive properties, PLGA particles can interact with the aorta’s macrophages and monocytes, resulting in improved drug absorption into systemic circulation due to controlled release [72].

#### Polyethylene glycol (PEG)-NPs

The surface modification of NPs with PEG, a process known as PEGylation, is a well-established strategy to improve the therapeutic efficacy and targeted delivery of drugs. PEG-NPs, which are typically approximately 50 nm in size, possess a hydrophilic surface layer that provides excellent biocompatibility and low toxicity [73]. These systems act as targeted vehicles that deliver anti-inflammatory mediators directly to VCAM-1 expressing dysfunctional endothelium, thereby blocking monocyte recruitment and secondary inflammatory responses. A novel nanosystem, VHPK-PLGA@COL, comprising colchicine encapsulated within PLGA and PEG, and decorated with the VHPK peptide (which can enhance targeting VCAM-1 on endothelial cells), was shown to enhance colchicine release while reducing its side effects. In preclinical studies, VHPK-PLGA@COL reduced atherosclerotic plaque formation by 30%, affecting the NF-κB/NLRP3 pathway, compared with that in the control group, and demonstrated favorable therapeutic effects and long-term safety [74]. Another PEGylated formulation, PEG-liposome-miR-146a, which carries the anti-inflammatory microRNA miR-146a, significantly inhibited endothelial and smooth muscle cell activation and foam cell formation while increasing the stability and delivery efficiency of miR-146a [51]. In summary, PEG-NPs represent a promising approach for enhancing therapeutic outcomes and improving the biosafety of drug delivery systems.

#### Polymeric micelles

Polymeric micelles are nanoscale drug delivery systems that typically range from 10 to 100 nm in size. They are composed of a hydrophobic core and a hydrophilic shell, which allows the encapsulation of hydrophobic drugs within the micellar core [75, 76]. Common synthesis methods include direct dissolution, dialysis, oil-in-water emulsion, and microfluidics [77]. Polymeric micelles offer several advantages, including high encapsulation efficiency, ease of preparation, prolonged drug retention, and excellent biocompatibility [78]. Sugar-derived amphiphilic macromolecule (AM) micelles, for example, can competitively bind to scavenger receptors to inhibit oxLDL uptake. By blocking these receptors, AM micelles inhibit oxLDL uptake by 85% and significantly reduce pro-atherogenic signaling in macrophages, smooth muscle cells, and endothelial cells. Notably, AM micelles inhibited oxLDL uptake by 85% and significantly reduced inflammatory signaling in macrophages, smooth muscle cells, and endothelial cells. These findings suggest that AM micelles may exert anti-atherogenic effects by attenuating inflammation [79].

### Biomimetic nanomaterials

Biomimetic nanomaterials have garnered significant interest as innovative drug delivery strategies. These materials primarily consist of NPs coated with biomimetic cell membranes (cell membrane-based NPs), including those derived from erythrocytes, macrophages, platelets, and monocytes. The incorporation of these membranes into NP designs enhances drug delivery by improving targeting capabilities and prolonging circulation time in the bloodstream, thereby increasing both the safety and efficacy of therapeutic agents [41].

#### Erythrocyte membrane-coated NPs

Erythrocytes are the most abundant cells in the blood, and mature erythrocytes lack various organelles and nuclei, making them easy to extract and purify. Their biconcave disc shape is advantageous for providing a coating material in drug delivery systems. [80] Erythrocyte membrane-coated NPs can be prepared in various sizes, typically ranging from 70 to 200 nm [81]. The primary mechanism for their prolonged survival is the presence of CD47, a membrane protein that binds to the SIRP-α receptor on macrophages to deliver a “don’t eat me” signal, which actively inhibits phagocytic clearance. This allows the particles to accumulate in atherosclerotic lesions through the enhanced permeability and retention effect caused by the leaky endothelium in inflamed vessels. These NPs, which encapsulate drugs or other therapeutic compounds, can be fabricated via physical or chemical methods, including erythrocyte membrane-derived vesicles, vesicle–particle fusion, membrane-templated approaches, and microfluidic electroporation [82]. As carriers, erythrocyte membrane-coated NPs exhibit prolonged circulation times, low toxicity, excellent biocompatibility and biodegradability, and a lifespan of up to 120 days. [80]. In one study, erythrocyte membrane-coated PLGA NPs loaded with rapamycin (RBC/RAP@PLGA) significantly extended the circulation time by 31% compared with that of the control groups [83]. Importantly, these NPs do not release the drug in healthy blood vessels but instead facilitate targeted delivery to atherosclerotic regions. In conclusion, the integration of erythrocyte membranes with other nanomaterials holds promise for achieving both passive and active targeted drug delivery.

#### Platelet membrane-coated NPs

Platelets are produced by mature megakaryocytes in the bone marrow. They are disc-shaped, nuclear cellular fragments with an average diameter of 2–4 μm and a lifespan of 7–10 days [84]. Platelet membrane-coated NPs, with an average size of approximately 130 nm, have been a focus of research over the past decade [85]. These NPs have attracted attention because of their immune evasion, endothelial adhesion, and targeted delivery capabilities. Their targeting ability is driven by a natural binding affinity for macrophage-derived foam cells and damaged vascular sites, leveraging adhesion molecules like P-selectin to bind to CD44 receptors on target cells. In addition to their role in hemostasis, platelet membrane-coated NPs have demonstrated therapeutic potential in atherosclerosis, cancer, and infectious diseases [86]. Platelet membranes were coated onto the surfaces of upconverting nanoparticles (UCNPs) and chlorin e6 (Ce6), which were encapsulated by polyacrylate-n-octyl amine (PAAO) micelles, forming a nanosystem named PM-PAAO-UCNPs. PM-PAAO-UCNPs exhibited strong red fluorescence signals in foam cells, but no signal in healthy intima. These findings indicate that PM-PAAO-UCNPs can specifically target foam cells following platelet membrane functionalization [87]. Furthermore, platelet membrane-wrapped NPs have been employed to deliver fluorescent photoresponsive NO prodrugs (RBT-NO), facilitating NO release for endothelial repair at plaque sites. Histological analysis revealed that the control group presented thickened vessel walls, a reduced lumen diameter, and intimal hyperplasia, whereas the treatment group presented a more regular vessel wall architecture and reduced thrombus accumulation [88]. Overall, platelet membrane technology holds promise for enhancing the precision of drug targeting and enabling early-stage detection of atherosclerotic plaques in the treatment of ASCVD.

#### Macrophage membrane-coated NPs

Macrophages play crucial roles in various stages of atherosclerosis and are classified into two phenotypes: the proinflammatory M1 phenotype and the anti-inflammatory M2 phenotype [89]. Macrophage membrane-coated NPs are composed of a thin layer of cell membrane encapsulating a NP core, resulting in structures approximately 90 nm in size [90, 91]. These NPs home to atherosclerotic plaques by utilizing integrins (such as α4 and β1) to recognize and bind to VCAM-1 overexpressed on the inflamed vascular endothelium. Furthermore, they employ a "nanosponge" mechanism where membrane receptors like TNFR2 and CCR2 act as decoys to sequester and neutralise circulating proinflammatory cytokines (e.g., TNF-α and IL-1β), thereby suppressing local inflammation. The macrophage membrane not only enhances the targeted delivery of NPs to atherosclerotic lesion sites but also serves as a scavenger for proinflammatory factors [92]. Macrophage membrane-coated atorvastatin NPs (MM-AT-NPs), demonstrating selective drug release in cells with elevated ROS production. Additionally, MM-AT-NPs significantly reduced the atherosclerotic lesion area by decreasing CD14⁺ macrophage infiltration and MMP-9 level in *ApoE*^⁻/⁻^ mice, suggesting that macrophage membrane-coated NPs constitute a promising platform for targeted drug delivery in atherosclerosis [93].

#### Monocyte membrane-coated NPs

During atherosclerosis progression, endothelial cells release chemokines and adhesion molecules to facilitate monocyte infiltration. Therefore, drugs coated with monocyte membranes have the potential to target atherosclerotic sites. The mechanism of action involves mimicking the inherent leukocyte recruitment pathways, allowing the nanoparticles to roll, adhere, and transmigrate through the vascular wall into the plaque core [94]. Monocyte membrane-coated NPs, which typically have an average size of less than 200 nm, improve cellular targeting, receptor binding, and intracellular uptake during disease progression [95]. Monocyte membrane-coated PLGA NPs delivering gliclazide were shown to reduce the atherosclerotic burden by 6.4-fold and decrease inflammation levels in rabbits fed a high-cholesterol diet [96]. Additionally, a monocyte cell membrane-coated 1,8-cineole biomimetic delivery system (MM-CIN-BDS) alleviated atherosclerosis by reducing the lesion area. MM-CIN-BDS also downregulated the expression of adhesion molecules and inflammatory mediators such as ICAM-1, VCAM-1, NF-κB, TNF-α, and PPAR-γ in the aortas of C57BL/6 mice fed an atherogenic diet [97]. Overall, monocyte cell membrane-coated NPs can more effectively target lesion sites, enhancing drug efficacy and reducing inflammation in atherosclerosis.

### Hybrid NPs

To mitigate the limitations of individual NPs, hybrid NPs-comprising at least two different types of NPs-can enhance the properties of single-component NPs and achieve multifunctionality [98]. Hybrid NPs may combine inorganic, organic, or biomimetic nanomaterials to form unique composites with synergistic capabilities [99]. For example, a hybrid membrane composed of macrophage and red blood cell (Mø-RBC) membranes was coated onto the surface of graphene oxide quantum dot (GOQD) NPs loaded with atorvastatin (AT) and further modified with HA, forming a nanosystem termed HA-M@AT@GP. HA-M@AT@GP, through pH-responsive release, reduced the levels of proinflammatory cytokines such as TNF-α, IL-1β, IL-6, and MCP-1 and decreased the plaque area by 30% in the *ApoE*^−/−^ mice [100]. Therefore, this hybrid NP strategy enables prolonged retention in inflammatory atherosclerotic plaques.

## Diagnostic imaging using nanoparticles

Current noninvasive diagnostic tools have a limited ability to discriminate between stable and vulnerable plaques. As molecular imaging technologies facilitate the high-resolution visualization of atherosclerotic plaques, nanomaterial-based imaging offers deep insights into plaque composition. In addition, nanomaterials exhibit enhanced bioavailability, biocompatibility, stability, and permeability, along with an optimal circulation time under physiological conditions. Currently, both passive and active targeting strategies are being explored for the diagnostic imaging of atherosclerosis (Table 2). Passive targeting in atherosclerosis is primarily driven by the ELVIS effect (Extravasation through Leaky Vasculature and Inflammatory cell-mediated Sequestration), a phenomenon analogous to the EPR effect in oncology [101]. However, its clinical utility is often limited by the high heterogeneity of human plaque biology and vascular permeability compared to highly controlled animal models [102]. Contrary to passive targeting, the active targeting strategy provides more precise drug delivery to atherosclerotic sites. It is designed to create nanocarriers with surface-modified ligands, enabling NPs to selectively recognize and bind to plaque [103]. VCAM-1–targeted NPs exhibited a 2.6-fold higher plaque targeting efficiency than passively targeted NPs in *ApoE*^−/−^ mouse models [104]. Nevertheless, the active targeting strategy still has limitations for imaging in ASCVD. One major limitation is the protein corona (PC) effect, which refers to the adsorption of proteins onto the NP surface and causes NPs to be cleared quickly by phagocytic cells in multiple organs, such as the lungs, spleen, and liver. It is resulting in reduced delivery efficiency, limited lesion site residence time, and non-specific imaging [105, 106].

**Table 2:** Diagnostic imaging strategies of nanoparticles for ASCVD

Type of targeting	Classification	NPs for diagnostic imaging	Model	Mechanosensing Contributors	References
Passive targeting	pH-sensitive	IONP-HP is response to the acidic environment of lysosomes in the vulnerability of plaques by using MRI	*ApoE*^−/−^ mice fed with HFD for 3 months in stable plaque group	CD14 and CD68 receptors expression	[108]
			*ApoE*^−/−^ mice fed with HFD for 12 months in vulnerable plaque group		
	ROS-sensitive	RSPNs for PAI is used to measure the level of •O^2−^ level in plaque	*ApoE*^−/−^ mice fed with HFD for 16 weeks	•O^2−^ level upregulation	[110]
	Lipid-specific	RBC/LFP@PMMP can bind with lipids in lipid abundant inflammatory plaques and emerge green fluorescence detected by the confocal laser scanning microscopy flow cytometry	*ApoE*^−/−^ mice fed with HFD for 9 weeks	oxLDL uptake; ROS overexpression; MMP-9 activity	[111]
	Enzyme-reactive	A cathepsin-B-activatable fluorescent liposomes probe (Peptide–ICG2) for the NIRF has been studied for imaging vulnerable atherosclerotic plaques	*ApoE*^−/−^ mice fed with HFD for 24 to 39 weeks	Cathepsin B expression; PS receptor highly expressed on the cell surface of the macrophages	[112]
			WHHL rabbit and normal New Zealand White fed with daily HFEQ and an alfalfa-free CR-3 diet daily		
Active targeting	Macrophage-targeted	Thiolated glycol chitosan with cholesteryl chloroformate and maleimide-PEG-mannose nanoprobe targeting MMR which was labeled by NIRF in carotid plaques	*ApoE*^−/−^ mice fed with HFD for 20 weeks	Mannose receptors expression on macrophages	[116]
			New Zealand white rabbits were operated by balloon denudation and then fed on a HFD for 8 weeks		
	Foam cell-targeted	The OPN targeting UCNPs probes specifically target foam cells in vitro by confocal fluorescence imaging	*ApoE*^−/−^ mice fed with HFD for 2 weeks prior to being do partial carotid ligation, the mice are continued to feed HFD for 16 weeks	OPN overexpression in macrophages and foam cells	[117]
	Inflammation-targeted	Fe_3_O_4_ nanoparticles modified with 5-HT and fluorescent dye Cy7, which can actively map MPO for targeting of vulnerable plaques by MRI, CT, and fluorescence imaging	*ApoE*^−/−^ mice fed with HFD for 39 to 41 weeks	MPO activity and expression	[120]
	Angiogenesis-targeted	Pluronic-based nanocarriers conjugated with cRGD peptide and collagen IV peptide, which targeted to ανβ3 integrin receptor for imaging of plaques in the early stage of atherosclerosis by MRI	*ApoE*^−/−^ mice fed with HFD for 8 to 12 weeks	ανβ3 integrin activation; collagen type IV content	[123]
	ECM-targeted	Synthesized AuNPs with high light-to-acoustic conversion efficiency to be used as a PAI probe for tracing MMP2	New Zealand white rabbits fed with HFD for 25 days first, then doing the balloon injury within the thoracic aorta. After the injury, rabbits are continued to feed HFD for 20 weeks	MMP2 activity and expression	[125]

IONP-HP: hyaluronic acid and poly(acrylic acid) (PAA)-modified iron oxide nanoprobe; MRI: magnetic resonance imaging; ApoE: apolipoprotein E; HFD: high fat diet; ROS: reactive oxygen species; RSPNs: ratiometric semiconducting polymer nanoparticles; PAI: photoacoustic imaging; O^2−^: superoxide anions; RBC/LFP@PMMP: a prodrug copolymer, PMPC-P(MEMA-co-PDMA) (PMMP), was assembled with LFPs and subsequently coated with an RBC membrane; ICG2: indocyanine green 2; NIRF: near-infrared fluorescence; WHHL-rabbit: Watanabe-heritable hyperlipidemic rabbit; PS: phosphatidylserine; PEG: polyethylene glycol; MMR: macrophage mannose receptor; OPN: osteopontin; UCNPs: upconversion nanoparticles; Fe_3_O_4_: ferri-ferrous oxide; 5-HT: 5-hydroxytryptamine; MPO: myeloperoxidase; CT: computed tomography; cRGD: cyclic-arginine-glycine-aspartic; ECM: extracellular matrix; AuNPs: gold nanoparticles; MMP2: matrix metalloproteinase 2

### Passive targeting imaging

Passive targeting is a diagnostic imaging strategy that delivers NPs directly to atherosclerotic plaques. NPs can accumulate in these plaques due to their high retention characteristics, which result from increased vascular permeability and openings in the endothelial lining of the affected blood vessels [101].

#### pH-sensitive imaging

The pH of vulnerable plaque lesion areas has been reported to be mildly acidic (approximately 5.5) because of microenvironmental changes such as hypoxia, lactate accumulation, and macrophage infiltration [107]. In a previous study, an HA and poly(acrylic acid) (PAA)-modified iron oxide nanoprobe (IONP-HP) was developed to achieve *T*_*1*_–*T*_*2*_ switchable contrast effects in acidic environments, enabling the noninvasive evaluation of plaque vulnerability via MRI. When modified with HA, IONP-HP specifically targets vulnerable, macrophage-rich plaques, as the probe is responsive to the acidic conditions of lysosomes. The study demonstrated that IONP-HPs presented a strong *T*_*1*_-enhanced signal in vulnerable plaques, which switched to a *T*_*2*_-enhanced signal after 9 h in *ApoE*^−/−^ mice. In contrast, only a slight *T*_1_ signal was observed in stable plaques, with no detectable *T*_*2*_ enhancement. [108] This work not only introduces a novel MRI nanoprobe with dual-mode switching capability but also highlights the potential of pH-responsive nanoprobes for detecting vulnerable atherosclerotic plaques.

#### Reactive oxygen species (ROS)-sensitive imaging

Early atherosclerotic lesions are often missed by imaging techniques such as echography and angiography, as the luminal diameter and vessel wall thickness typically show minimal changes compared with those of normal arteries. Since early-stage lesions are characterized by oxidative stress and inflammatory responses, novel tools capable of detecting atherosclerosis via ROS formation could facilitate the identification of these early pathological changes [109]. In vulnerable plaques, substantial levels of superoxide anions (•O₂⁻) and hydrogen peroxide (H₂O₂) are produced in response to inflammation, contributing to cellular apoptosis. The development of ratiometric semiconducting polymeric nanoparticles (RSPNs) that respond to •O₂⁻ has enabled in vivo PAI of vulnerable atherosclerotic plaques. RSPNs leverage the differential PA signal intensities generated by a •O₂⁻–responsive molecule and a •O₂⁻-insensitive molecule when excited at wavelengths of 690 nm and 800 nm, respectively. The PA₆₉₀/PA₈₀₀ ratio of RSPNs is positively correlated with the •O₂⁻ concentration, and plaques exhibiting significantly elevated PA₆₀/PA₈₀₀₀ ratios are at increased risk of rupture [110]. Therefore, the PA_690_/PA_800_ signal ratio can serve as a quantitative indicator of ROS levels and a biomarker for atherosclerotic plaque progression.

#### Lipid-specific imaging

Recently, a novel method utilizing lipid-specific fluorophores (LFPs) and fluorophore-labeled NPs, a prodrug copolymer, PMPC-P(MEMA-co-PDMA) (PMMP), was assembled with LFPs and subsequently coated with an RBC membrane, forming a biomimetic nanosystem referred to as RBC/LFP@PMMP, which has been investigated for the diagnosis of atherosclerosis [111]. When dissolved in water, LFPs emit strong orange fluorescence at a wavelength of 580 nm, whereas dissolution in oil results in a blueshift of the emission wavelength to 525 nm, producing green fluorescence. Thus, in lipid- and ROS-rich inflammatory plaques, LFPs can bind to lipids and exhibit green fluorescence in response to excessive ROS production. LFPs not only offer a lipid-specific imaging strategy but also serve as a potential delivery platform for endogenous lipids to facilitate controlled drug release [111].

#### Enzyme-reactive imaging

Under inflammatory conditions, activated macrophages release proteases, such as cysteine proteases and MMPs, into the plaque microenvironment, which promotes collagen fragmentation and fibrous cap degradation, thereby increasing the risk of plaque rupture [107]. Consequently, elevated protease levels within plaques constitute a critical feature for the development of enzyme-reactive strategies for plaque imaging. Recently, a cathepsin B-activatable fluorescent probe, peptide–indocyanine green 2 (ICG2), has been investigated for near-infrared fluorescence (NIRF) imaging of vulnerable atherosclerotic plaques [112]. Cathepsin B, a cysteine hydrolase, is highly expressed in the lysosomes of macrophages. The linker peptide of ICG2 is cleaved by cathepsin B, triggering fluorescence emission and enabling the specific detection of macrophages within plaques. In an in vivo study, *ApoE*^⁻/⁻^ mice treated with liposomes-peptide-ICG2 presented the strongest fluorescence signal localized in the lesion area [112]. Therefore, enzyme-reactive NIRF probes offer the advantage of reducing nonspecific background noise, thereby allowing more precise imaging of plaque vulnerability.

### Active targeting imaging

Passive targeting is susceptible to the physicochemical properties of NPs, often resulting in nonspecific imaging and reduced targeting efficiency. As such, NPs functionalized with ligands or biomarker-specific moieties can help actively and precisely target atherosclerotic plaque lesions [113]. Within the microenvironment of vulnerable plaques, specific biomarkers associated with macrophages, foam cells, inflammation, angiogenesis, and extracellular matrix (ECM)-rich regions have been investigated for their potential in active targeted imaging.

#### Macrophage-targeted imaging

Macrophage phenotypes are closely associated with the development and progression of atherosclerotic plaques. Imaging strategies targeting macrophages have been extensively employed in the diagnosis of ASCVD [114]. Several contrast agents and probes based on macrophage-targeting NPs, including those compatible with positron emission tomography (PET), computed tomography (CT), and magnetic resonance imaging (MRI), have been investigated for their ability to track macrophage recruitment to atherosclerotic plaques [115]. One such example is a macrophage mannose receptor (MMR)-targeted nanoprobe labeled with mannose and the fluorescent dyes cyanine5.5 and cyanine7, which exhibit significantly enhanced NIRF in carotid plaques. This signal correlated with fluorescence imaging, Oil Red O (ORO) staining, and hematoxylin and eosin (H&E) staining in atherosclerotic lesions in vivo [116]. Macrophage-targeted imaging plays a crucial role in diagnostic strategies by enabling the identification of vulnerable plaque risk through tracking macrophages in atherosclerotic lesion areas.

#### Foam cell-targeted imaging

Foam cell formation is a central pathological process in the development of atherosclerosis. Owing to the cytotoxicity and immunogenicity of ox-LDL, its uptake leads to foam cell necrosis, ultimately resulting in vulnerable plaque formation [107]. Therefore, targeting foam cells is a critical strategy for imaging atherosclerotic plaques. Osteopontin (OPN), a glycosylated phosphoprotein, is overexpressed in foam cells derived from macrophages and VSMCs compared with normal arterial walls and contributes to the destabilization of atherosclerotic plaques [107]. OPN-targeted upconversion nanoparticle (UCNP) probes have demonstrated that OPN-specific UCNPs can selectively bind to foam cells in vitro. Moreover, these UCNPs preferentially accumulate in vulnerable plaques of the carotid artery [117]. In summary, targeting foam cells represents a promising strategy for the detection of atherosclerotic plaques, particularly those that are vulnerable.

#### Inflammation-targeted imaging

Multiple inflammatory cells and proinflammatory factors are involved in the pathophysiology of atherosclerosis [118]. Activated macrophages and neutrophils can release myeloperoxidase (MPO), a heme peroxidase, and its oxidative products are related mainly to plaque vulnerability [119]. Fe_3_O_4_ NPs modified with 5-hydroxytryptamine (5-HT) and the fluorescent dye Cy7 selectively targeted MPO to visualize vulnerable atherosclerotic plaques. In addition, MRI, CT, and fluorescence imaging revealed greater signal intensity in plaques in *ApoE*^−/−^ mice than in control mice, which is consistent with the histological results. [120] Therefore, inflammation-related markers can be key targets for imaging vulnerable atherosclerotic plaques.

#### Angiogenesis-targeted imaging

In response to inflammation and hypoxia, atherosclerotic plaques exhibit a compensatory mechanism involving neovascularization. Angiogenesis within plaques is associated with an increased risk of plaque rupture [121]. Therefore, neovascularization serves as a key target for assessing plaque vulnerability. Several biomarkers, including vascular endothelial growth factor (VEGF), integrin αvβ3, and hypoxia-inducible factor (HIF), are reported to play roles in angiogenesis [122]. Pluronic-based nanocarriers conjugated with cyclic-arginine-glycine-aspartic (cRGD) peptide and collagen IV peptide can bind to the αvβ3 integrin receptor via the cRGD peptide, enabling more efficient targeting for the imaging detection of the early stage of atherosclerotic plaques [123].

#### Extracellular matrix (ECM)-targeted imaging

The ECM, which consists mainly of collagen during the formation of the fibrous cap in atherosclerotic plaques, plays a crucial role in maintaining plaque integrity. During the development of atherosclerosis, apoptotic VSMCs release MMPs, leading to ECM degradation and thinning of the fibrous cap. Therefore, MMPs are considered key biomarkers for imaging vulnerable plaques [124]. Synthesized AuNPs with high light-to-acoustic conversion efficiency have been used as PAI probes conjugated with an MMP-2 antibody. This nanoprobe enables the measurement of MMP-2 levels and facilitates the identification of high-risk plaques through intravascular PAI [125]. In summary, mapping MMPs represents a promising NP-targeted ECM-based imaging strategy, supporting further development of NP-based photoacoustic methods for the clinical diagnosis and treatment of atherosclerosis. In addition to NP-targeted imaging for the diagnosis of atherosclerosis, nanomedicine can also target specific cellular and microenvironmental components to enhance treatment efficacy, reduce side effects, and enable precise drug delivery to pathological sites.

## Targeted nanomedicine therapeutics for ASCVD

**Fig. 2:**
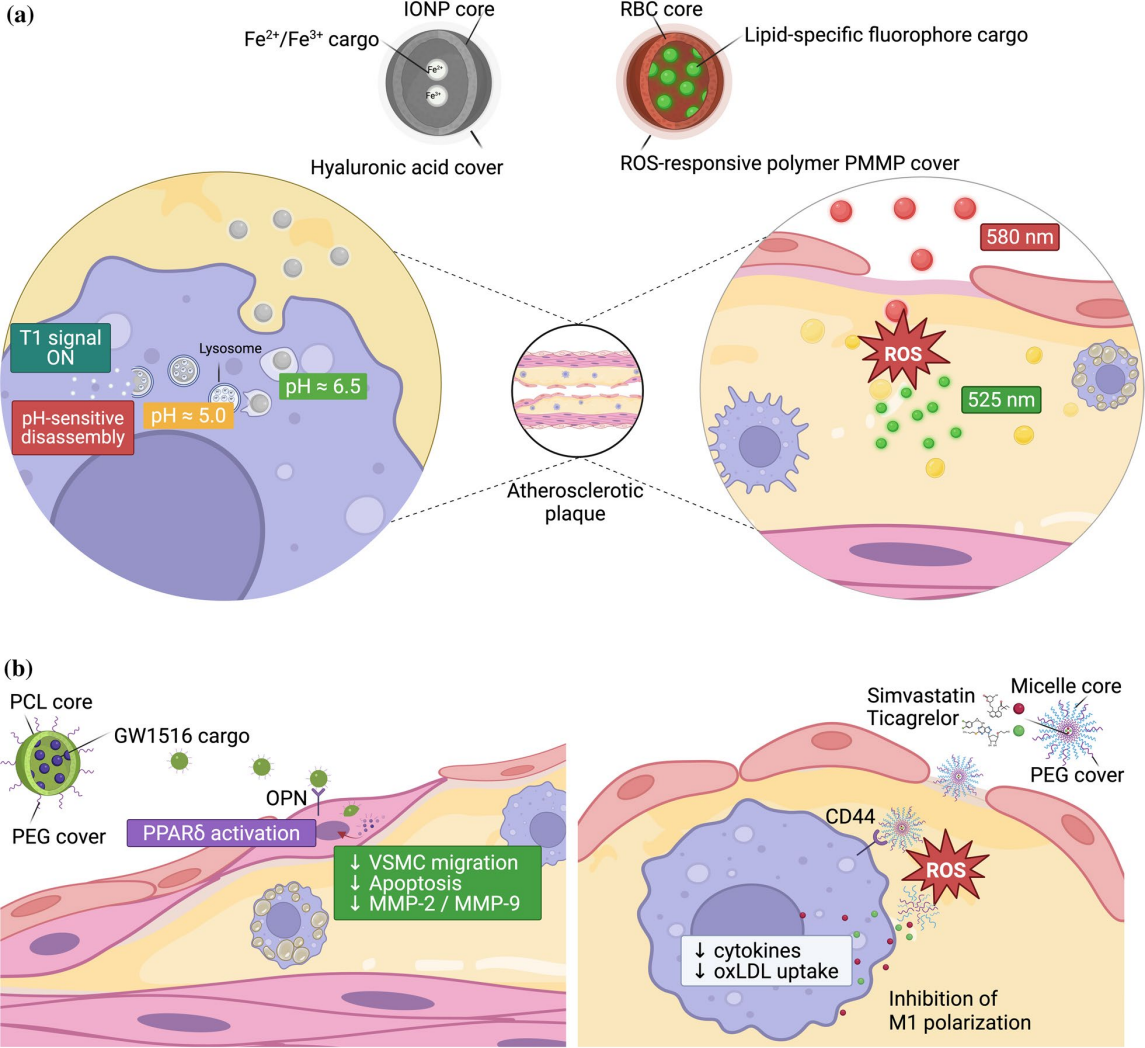
Precision nanomedicine diagnostic and therapeutic strategies for atherosclerotic cardiovascular diseases. **A** Diagnostic nanoparticles. (Left) pH-sensitive magnetic resonance imaging (MRI) probes based on iron oxide nanoparticles (IONPs) use hyaluronic acid-mediated targeting of cell surface glycoprotein CD44-positive macrophages and accumulate within lysosomes, where acidic conditions induce membrane destabilization and activate a T1-switchable contrast signal. (Right) Biomimetic red blood cell (RBC)-derived nanoparticles incorporating a PMPC-P(MEMA-co-PDMA) copolymer (PMMP)-coated fluorophore enable reactive oxygen species (ROS)-responsive imaging, undergoing a ratiometric wavelength shift upon exposure to oxidative stress, thereby allowing precise visualization of lipid-rich, inflamed plaques. **B** Therapeutic nanoparticles. (Left) Targeted delivery systems of polycaprolactone (PCL)-core, polyethylene glycol (PEG)-covered nanoparticle directed at vascular smooth muscle cells (VSMCs) utilize osteopontin (OPN)-binding nanoparticles to selectively deliver the peroxisome proliferator-activated receptor-δ (PPAR-δ) agonist GW1516 to synthetic VSMCs, restoring contractile marker expression and reducing matrix metalloproteinase (MMP)-mediated extracellular matrix degradation. (Right) ROS-responsive polymeric micelles exploit CD44-mediated endocytosis in plaque-associated macrophages to deliver a dual payload of simvastatin and ticagrelor. Elevated ROS levels within the plaque microenvironment trigger bond cleavage and controlled drug release, leading to macrophage reprogramming characterized by suppression of proinflammatory M1 signaling and decreased oxidized low-density lipoprotein (oxLDL) uptake, ultimately resulting in significant lesion regression

**Fig. 3:**
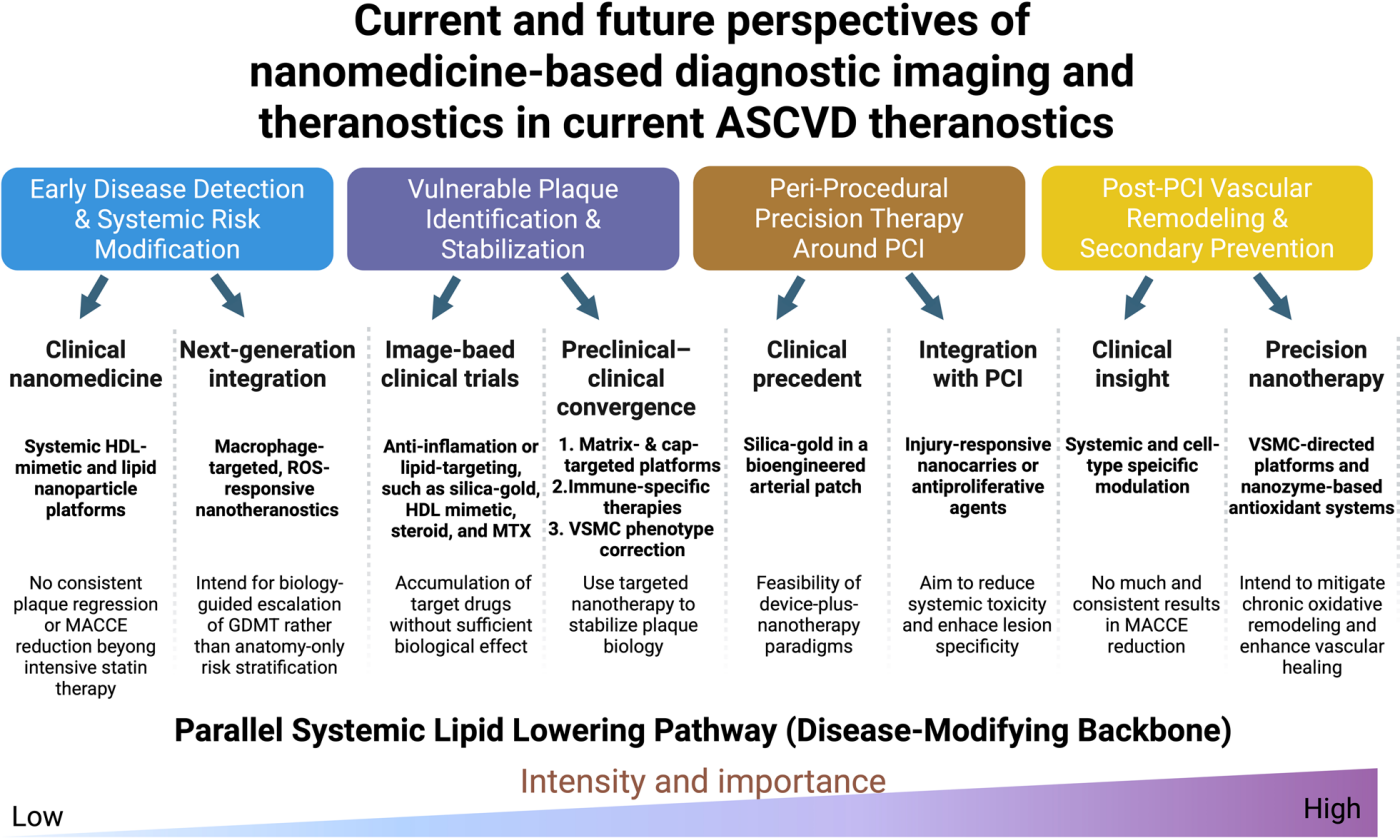
Current and future perspectives of nanomedicine-based diagnostic imaging and theranostics in ASCVD. The schematic summarizes four translational stages of nanomedicine development, spanning early disease detection and systemic risk modification, vulnerable plaque identification and stabilization, peri-procedural precision therapy around percutaneous coronary intervention (PCI), and post-PCI vascular remodeling and secondary prevention. Representative approaches include systemic HDL-mimetic or lipid nanoparticle platforms, image-guided plaque-targeted therapies, injury-responsive nanocarriers integrated with PCI, and vascular smooth muscle cell–directed antioxidant nanozyme systems. While early systemic strategies have shown limited plaque regression or MACCE reduction, later-stage approaches emphasize lesion-specific targeting and device-integrated nanotherapy to improve vascular healing. A parallel systemic lipid-editing pathway is depicted as a disease-modifying backbone across stages, with the gradient indicating increasing therapeutic intensity and clinical relevance. Abbreviation: PCI, percutaneous coronary intervention; HDL, high-density lipoprotein; MACCE, major adverse cardiac and cerebrovascular events

The clinical treatment of ASCVD is increasingly shifting toward theranostic approaches, where pathological features used for high-resolution imaging also serve as triggers for targeted treatment. As shown in Fig. 3, leading nanoplatforms exploit the unique microenvironment of vulnerable plaque, such as acidosis and the oxidative niche, to achieve both diagnostic accuracy and therapeutic efficacy. While switchable contrast and ratiometric shifts of fluorophores enable the identification of high-risk areas (Fig. 2A), these metabolic signals are simultaneously used to release beneficial substances for vascular smooth muscle cell stabilization and immune reprogramming (Fig. 2B). By integrating these diagnostic data into therapeutic design, nanomedicine facilitates an adaptive approach to atherosclerosis detection and plaque regression.

Given the unique pathophysiological and mechanical environments of atherosclerotic plaques, nanomedicine approaches aim to improve the therapeutic effect on lesions while minimizing systemic side effects. Like diagnostic strategies, both passive and active targeted therapies have been designed for the treatment of ASCVD (Table 3). These strategies are categorized by their intervention framework: either preventing plaque initiation by targeting circulating monocytes or promoting the regression and stabilization of established, vulnerable lesions through the modulation of the ECM. Mechanotransduction concepts are integrated into these platforms by targeting of ECM components, such as type IV collagen and fibronectin, which become pathologically exposed during endothelial injury and serve as high-affinity anchors for dual-responsive NPs. Furthermore, active targeting of osteopontin, which is overexpressed in synthetic-phenotype VSMCs, allows for the precise modulation of cell migration and phenotypic switching, directly addressing the root causes of plaque instability. Preclinical evidence underscores that NP design must be tailored to the specific evolutionary stage of the disease. For instance, stimuli-responsive hybrids can be engineered to exploit the acidic pH and elevated ROS levels characteristic of the necrotic cores, where oxidants, excess lipids, and inflammatory cells, as well as macrophage membranes and vascular smooth muscle cells serve as potential therapeutic targets. This section reviews advanced therapeutic nanotechnologies and illustrates how each strategy enhances drug localization, efficacy, and safety in preclinical models of ASCVD.

**Table 3:** Targeted nanomedicine therapeutics for ASCVD

Platform / Core (Cargo)	Targeted strategy and Mechanism	Interventional stage	Repeated Dosing & Clearance	Advantages and Clinical Barriers	Mechanosensing Contributors	Model	Pre-clinical (or Headline) Efficacy	References
HA–phenyl‑boronate micelle / (Methotrexate)	Active targeting (Integrin $\upalpha 4\upbeta 1$/VCAM-1) + ROS-responsive release	Established plaque (30-day treatment in HFD mice)	Every 3 days; accumulates in filtering organs (liver/kidney)	Adv: Avoids phagocytosis; inherent chemotactic targeting Limit: Complex membrane extraction	Integrin expression; Collagen content	HUVEC, RAW264.7, *ApoE*^⁻/⁻^	− 45% plaque area	[131]
Fibronectin‑targeted thioketal/(Simvastatin + ticagrelor)	CREKA peptide for fibrin-rich lesions + ROS-cleavable linker	Progression/Established (8-week treatment during HFD)	Twice weekly; prolonged circulation via ROS-responsive PEG core	Adv: Synergetic anti-inflammatory co-delivery; high stability in circulation Limit: Statin therapy side effects	Fibronectin anchoring; TRPV4 oxLDL uptake as an indicator	ApoE^⁻/⁻^	↓ ROS, ↓ cytokine ↓Necrotic core area	[132]
HA‑PPS micelle (Simvastatin)	CD44-positive macrophage targeting + H_2_O_2_ scavenging by polymer core	Established (4-week treatment after 8-week HFD)	Once weekly; targeted via CD44-mediated endocytosis	Adv: Inherent antioxidant activity of carrier Limit: Statin therapy side effects	CD44-HA interactions; Plaque cholesterol as an indicator	RAW264.7, *ApoE*^⁻/⁻^	↓ ROS, aortic plaque lesions	[133]
HA‑CeO₂ nanozyme/ (Catalytic antioxidant)	Active targeting of CD44^+^ macrophages via HA shell	Initiation to progression	Every 3 days; high liver/spleen accumulation	Adv: Reversible self-healing redox cycling for constant ROS consumption Limit: Potential accumulation in liver/spleen	Hyaluronidase activity; SOD-mimic activity	*LDLR*^⁻/⁻^	− 35% lesion ↓ serum LDL	[5]
β‑cyclodextrin dual pH/ROS (Rapamycin)	Col-IV peptide targeting + Acidosis and ROS triggered disassembly	Acute injury (restenosis model)	Twice weekly; renal/hepatic clearance	Adv: Precise triggered release only in acidic/oxidative microenvironments Limit: Complex pharmaceutical scale-up and reproducibility	MMP-2 activity; Col-IV binding	Sprague–Dawley rats, C57BL/6 J, MOVAS cells	Anti-restenosis efficacy ↓ Proliferation index	[135]
OPN‑peptide (GW1516)	Osteopontin in synthetic VSMCs	Regression (2-week treatment after 16-week HFD)	Four times weekly; sustained release and long circulation	Adv: Specifically corrects abnormal VSMC migration and apoptosis Limit: GW1516 carries systemic cancer risk in rodents	FAK phosphorylation; OPN content	MOVAS cells, *ApoE*^⁻/⁻^	− 30% plaque	[145]
miR‑145 micelle (miR‑145)	MCP-1 peptide targeting/VSMC pathways	Chronic disease	Repeated dosing possible; peptide-amphiphiles are biodegradable	Adv: Rescues contractile function across all disease severities Limit: miR delivery relies on qualitative disease staging	ACTA2 / MYH11 (Contractility)	Human VSMCs	Restore contractile function	[146]
HDL‑mimic (CD40–TRAF6 inhibitor)	ApoA-I monocyte homing + competitive signaling inhibition	Initiation and established	Twice weekly; macrophage-specific uptake in liver/spleen	Adv: inhibits plaque-driving inflammation without systemic immune suppression Limit: High liver/spleen uptake requires nanotherapeutic precision	$\upbeta$ 2**—**integrin expression	*ApoE*^⁻/⁻^, *CD40*^⁻/⁻^	− 25% plaque, plaque stabilization	[141]

ACTA2: α-smooth muscle actin (contractile marker); ApoA-I: apolipoprotein A-I; ApoE: apolipoprotein E; β-CD: β-cyclodextrin; CD40: cell surface receptor; CD44: cell-surface glycoprotein 44 (hyaluronic acid receptor); CeO₂: cerium oxide; Col-IV: type IV collagen; CREKA: Cys-Arg-Glu-Lys-Ala (fibrin-binding peptide); FAK: focal adhesion kinase; GW1516: PPAR-δ agonist cargo; HA: hyaluronic acid; H₂O₂: hydrogen peroxide; HDL: high-density lipoprotein; HFD: high-fat diet; HUVEC: human umbilical vein endothelial cell; IONP: iron oxide-based nanoparticle; LDLR: low-density lipoprotein receptor; MCP-1: monocyte chemoattractant protein-1; miR: microRNA; miR-145: microRNA-145; MMP: matrix metalloproteinase; MOVAS: mouse aortic vascular smooth muscle cell line; MYH11: myosin heavy chain 11; OPN: osteopontin; oxLDL: oxidized low-density lipoprotein; PEG: polyethylene glycol; PPAR: peroxisome proliferator-activated receptor; PPS: polypropylene sulphide; RAW264.7: murine macrophage cell line; RBC: red blood cell; ROS: reactive oxygen species; SOD: superoxide dismutase; TRAF6 – TNF (tumor necrosis factor) receptor-associated factor 6; TRPV4: Transient receptor potential cation channel subfamily V member 4: VCAM-1: vascular cell-adhesion molecule-1; VSMC: vascular smooth muscle cell

### Passive targeted therapies

#### pH-sensitive therapies

Periplaque acidosis, which enables imaging, can be repurposed as an onboard signal for drug release [126, 127]. A growing toolkit of acid-sensitive carriers releases their payloads only when they have reached diseased tissue. HA micelles stitched together by hydrazone bonds protonate and disassemble in mildly acidic fluid, co-releasing all-trans-retinal and rapamycin to quench ROS, suppress the tumor necrosis factor TNF-α, and regress aortic plaques in *ApoE*^*−/−*^ mice [128]. Losartan-loaded zeolitic imidazolate framework ZIF-8 dissolves when protons break Zn-imidazolate linkages, delivering both antihypertensive drugs and Zn^2^⁺ ions that synergistically reduce inflammation and lipid deposition [129]. Poly(β-amino-ester) micelles become protonated below pH 6.8, switch polarity, fall apart, and liberate shSiglec-1, reprogramming macrophages toward a “cold” phenotype that regresses advanced lesions [130]. Together, these platforms show how acid-cleavable linkers, metal–ligand dissociation, and proton-induced phase inversion can be tuned to the biochemical niche of vulnerable plaques, achieving spatially precise delivery of small molecules or nucleic acids and highlighting intraplaque acidosis as a powerful trigger for preclinical anti-atherosclerotic nanomedicine.

#### ROS-sensitive therapies

Prooxidative stress is a molecular hallmark of vulnerable plaques, making ROS an attractive trigger for precise drug release. A growing toolbox of ROS-reactive carriers now spans every major material class. Organic systems such as HA phenyl-boronate micelles harness H₂O₂-specific bond scission to liberate methotrexate, curb oxLDL uptake, and reduce macrophage inflammation in *ApoE*^*−/−*^ mice [131]. Thioketal prodrug NPs self-assemble around simvastatin and ticagrelor, and plaque H₂O₂ cleaves the linker, unleashing a triple payload that repolarizes M1 macrophages, lowers cytokines, and shrinks lesions by half [132]. Oxidation-sensitive PEG-poly(tyrosine-ethyl oxalyl) (PEG-Ptyr) micelles switch from hydrophobic to hydrophilic when their tyrosine-oxalate cores react with ROS, simultaneously scavenging peroxides and releasing simvastatin via CD44-targeted uptake [133]. Collectively, none of these platforms have yet entered first-in-human studies, but their capacity to convert an endogenous stress signal into site-restricted therapy positions ROS-responsive nanomedicine as a logical next step toward clinically meaningful plaque-selective intervention.

#### Hybrid-sensitive therapies

Hybrid‐responsive nanomedicines are now pushing beyond the new trigger paradigm by wiring multiple plaque cues into a single, self-reporting construct. A prime example is a photoacoustic/fluorescent NP built around an acid- and ROS-labile polyphenol scaffold. Only when both signals are present does the carrier unlock its cargo-atorvastatin, an antioxidant co-factor and a cholesterol-crystal solvent, while its optical readout simultaneously maps drug release in vivo, thereby decreasing atherosclerotic lesions [134]. A related strategy involves the same pH/ROS chemistry as a collagen-IV–homing shell; exposure to the inflamed, degraded basement membrane triggers on-site rapamycin delivery and sharply curbs smooth muscle hyperplasia after angioplasty in rats [135]. Mechanical stimuli can also be layered on these surfaces. RBC-hitchhiking vesicles detach in low-shear bifurcations and then exploit plaque ROS to liberate astaxanthin and quench oxidative stress [136]. Finally, a lipase-cleavable lipid core nested inside a pH/ROS-sensitive shell drives cholesterol efflux, whereas its built-in fluorophore flags the earliest lesions for intervention [137]. These multiplexed designs confer three strategic gains: tighter spatial precision by demanding coincidence of chemical and biomechanical cues, real-time confirmation of payload deployment through intrinsic imaging, and the ability to synchronize lipid removal, anti-inflammatory therapy, and antioxidant protection within a single dose. Although they involve greater synthetic complexity and a heavier regulatory burden, dual- and triple-responsive theranostic NPs offer clinicians a truly adaptive tool capable of simultaneously treating and visualizing plaques in real time within their evolving microenvironment.

### Lipid-directed therapies

Several groups now exploit the biochemical hallmarks of lipid-rich plaques—oxLDL, crystalline cholesterol, and lipase activity—to trigger site-restricted drug release. Sugar–cholesterol amphiphiles sterically cloak scavenger-receptor A1, preventing oxLDL uptake and foam-cell formation in *ApoE*^*−/−*^ mice [138], and phosphatidyl­serine vesicles adsorb directly to cholesterol crystals, dissolve them, and dampen NLRP3 signaling in *Ldlr*^*−/−*^ models [139]. rHDL particles bearing an LXR agonist amplify cholesterol efflux while attenuating local inflammation [133], whereas lipase-cleavable cholesteryl ester nanocapsules release atorvastatin only inside enzyme-rich plaques, reducing the lesion area by almost half [140]. Together, these platforms show that cholesterol can serve as a target, allowing simultaneous lipid depletion and anti-inflammatory action with minimal systemic exposure.

### Monocyte‒macrophage precision therapies

Because lesional macrophages orchestrate recruitment, cytokine release, and defective efferocytosis, multiple nanosystems are tailored to their biology. HDL mimics that ferry CD40 and release the tumor necrosis factor receptor-associated factor 6 (TRAF6) inhibitor curb Ly6C^high^ monocyte entry and strengthen the fibrous cap [141]. pH/ROS-sensitive HR_RAP_ micelles co-deliver all-trans-retinal and rapamycin to suppress oxidative bursts and halt macrophage proliferation [128]. Dual-payload particles carrying a Src homology region 2 domain-containing phosphatase-1 (SHP-1) blocker plus IL-10 increase efferocytosis and switch plaques to a resolving phenotype [142]. A rare-earth nanoconjugate of the single-chain variable fragment (scFv) novel antibody ASA6 both images and limits oxLDL internalization [143]. Enzyme-cleavable peptide amphiphiles that release the glucocorticoid annexin A1 protein-derived peptide Ac2—26 on MMP2/ROS cues provide an additional route to pro-resolving signaling [144]. Although still confined to rodent models, these examples highlight how macrophage-centric designs can be layered-recruitment blockade, proliferation control, lipid handling, and immuno-resolution to produce additive plaque stabilization.

### Smooth muscle‐targeted therapies

Phenotypic drift of VSMCs from contractile to synthetic forms fuels neointimal growth and fibrous cap weakening. A β-cyclodextrin network whose boronate and acetal bridges cleave only under acidic, oxidant-rich conditions liberates rapamycin precisely where collagen IV is exposed, halving restenosis in a rat carotid injury model [135]. OPN-directed NPs loaded with the PPAR-δ agonist GW1516 attenuate migration, MMP activity, and TGF-β/FAK signaling in *ApoE*^*−/−*^ mice [145]. Finally, PEG-PLA micelles that shuttle miR-145 restore contractile markers in patient-derived VSMCs and shrink plaques in *Ldlr*^*−/−*^ mice [146]. These approaches highlight the potential of VSMC-specific nanotherapeutics to mitigate vascular remodeling, decrease stenosis, and contribute to plaque stabilization.

### Gene-editing therapies

The precise rewriting of specific CV loci has shifted from proof-of-principle to preclinical reality, largely because NP vectors can shield fragile nucleic acid payloads and chaperone them to hepatocytes, endothelium, or lesional macrophages. Lipid NPs now carry adenine-base editors that install a single A → G conversion in PCSK9, driving sustained LDL-cholesterol lowering for weeks in mouse and nonhuman primate models after one systemic dose [147]. A similar CRISPR formulation with specific endothelial uptake was used to knock out the mechanosensitive gene thioredoxin domain containing 5, thereby stabilizing the eNOS protein and suppressing plaque formation [15]. Viral shells have kept pace: the hepatic adeno-associated virus AAV8-CRISPR cassette partially restores LDL-receptor activity in a familial hypercholesterolaemia model and reduces aortic lesions [148], whereas macrophage-derived exosomes ferry proresolving microRNAs that reset hematopoietic output and attenuate aortic inflammation [149]. Collectively, these studies illustrate the feasibility of editing strategies—base substitution, frameshift knockout, and epigenetic reprogramming—for delivering nanocarriers in vivo [150–152]. The therapeutic prospect is straightforward: a one-off edit could supplant lifelong statins or anti-cytokine injections and open previously undruggable nodes in lipid and inflammatory networks. In contrast to small-molecule delivery, LNP-mediated gene editing, such as PCSK9 targeting, is a highly infrequent treatment, aiming for permanent or long-acting modification, which can be safer than the systemic risk factors of lifelong medication. However, several challenges hinder clinical translation, including off-target cleavage, innate immune sensing of CRISPR components, large-scale manufacture of clinical-grade lipid or viral vectors, and the unique safety concerns associated with permanent genomic alterations.

### Major nanoparticle platforms in ASCVD

While inorganic NPs offer superior imaging capabilities, organic and lipid-based NP platforms currently dominate clinical translation due to their established biosafety profiles and scalable manufacturing potential. Emerging biomimetic and hybrid NPs aim to bridge these gaps by combining natural immune-evasive properties with synthetic versatility, albeit at the cost of increased manufacturing complexity (Table 4). Nanomedicine design should focus on the specific stage of the disease and its condition. Current preclinical platforms vary from every three days (HA-phenyl-borate micelles) to twice weekly (thioketal-simvastatin NPs). Optimizing intervention timing, dosing strategies, NP design, and efficacy validation are critical for the successful clinical translation of nanomedicine-based imaging approaches for ASCVD. (Table 5). Coronary Computed Tomography Angiography (CCTA) could identify and quantify calcified, non-calcified, and low-density necrotic core components, allowing the identification of high-risk plaque phenotypes. It has been shown to enhance the prediction of clinical cardiovascular events in high-risk populations [153, 154]. PET/CT imaging using 18F-sodium fluoride (18F-NaF) assesses microcalcification and macrophage activities, while CCTA could identify high-risk plaque phenotypes [155, 156]. Photon-Counting Detector Computed Tomography (PCD-CT) is an emerging advanced CT technology and introduces substantial technological improvements over conventional CT, including spatial and contrast resolution, energy discrimination, and reduction of various artifacts. This enables superior visualization and characterization of coronary atherosclerotic plaques, unravelling new phenotypes of plaque [157, 158]. Moreover, Coronary Magnetic Resonance Angiography (CMRA) using molecular magnetic resonance (MR) probes enables targeted imaging to visualize endothelium, extracellular matrix, macrophages, neovascularization, fibrin, proteolytic enzymes and lipids at all stages of atherosclerosis. In addition, MR offers a significantly higher spatial resolution, improved soft tissue contrast, and avoids ionizing radiation [159]. Optical Coherence Tomograph (OCT) provides a high-resolution, intracoronary imaging modality capable of visualization of coronary composition, including calcification, fibrous cap thickness, and macrophage abundance within atherosclerotic plaques [160, 161].

**Table 4:** Comparison of major nanoparticle platforms in ASCVD

Classification of NPs	Targeting efficiency	Stimuli-responsiveness	Imaging compatibility	Biosafety	Manufacturability /Batch Variability	Clinical readiness
Inorganic NPs (e.g. Metallic/ nonmetallic)	Passive (ELVIS effect); Active (ligand functionalization)	pH (IONP-HP); ROS (nanozymes)	To target vulnerable and macrophage-rich plaques due to acidic condition (IONP-HP)	Metallic NPs require coating to avoid cytotoxicity; Low toxicity for non-metallic NPs	High (easier to synthesize consistently at scale)	• First-in-human NANOM-FIM clinical trial (silica-gold)
Organic NPs (e.g. LNPs, liposomes, HDL-/ LDL-like)	Passive (ELVIS effect); Active (receptor-mediated)	pH (acid-labile lipids); Enzyme (hydrolase-cleavable)	To bind with lipids in lipid-rich plaques (RBC/LFP@PMMP); To specifically detect macrophages within plaques (ICG2-NPs)	High biocompatibility (but lipids may cause inflammation)	Moderate (high production costs but established methods (microfluidics))	• FDA approval (Inclisiran) • Phase Ib trial (VERVE-102) • Phase II/III trial (paclitaxel-loaded and methotrexate-loaded LDE)
Polymeric NPs (e.g. Natural, PLGA, PEG, micelles)	Active (peptide/ligand-decoration, e.g.VHPK-peptide)	pH/ROS; Enzyme	Respond to •O₂⁻ of vulnerable plaques (RSPNs)	Excellent for natural/PLGA (avoid phagocytosis)	High for micelles (but complex for multi-responsive scale-up)	• FDA approval (PLGA) • Phase I/IIa clinical trials for limb ischemia (NK-104-NPs)
Biomimetic NPs (Cell-membrane coated)	Active (membrane receptors for VCAM-1, foam cells)	Various cells-targeted	Can selectively bind to foam cells which accumulate in plaques (UCNPs)	Superior (but immune evasion, long circulation, and "self" recognition)	Low (complex membrane extraction and fusion processes)	Preclinical (limited mostly to rodent and rabbit models)
Hybrid NPs (Combined nanomaterials)	Multiplexed Active (dual-ligand shells)	Multisensitivity (pH + ROS)	Dual-mode (pH + ROS)	Optimized (intended to mitigate liabilities of individual classes)	Low (highest synthetic complexity and regulatory burden)	Preclinical (high complexity)

ELVIS: extravasation through leaky vasculature and subsequent inflammatory cell–mediated sequestration effect; IONP-HP: hyaluronic acid and poly(acrylic acid) (PAA)-modified iron oxide nanoprobe; ROS: reactive oxygen species; RBC/LFP@PMMP: a prodrug copolymer, PMPC-P(MEMA-co-PDMA) (PMMP), was assembled with LFPs and subsequently coated with an RBC membrane; ICG2: indocyanine green 2; LDE: lipid-nanoemulsion coating; PLGA: poly(lactic-co-glycolic acid); PEG: polyethylene glycol; RSPNs: ratiometric semiconducting polymeric nanoparticles; NK-104-NPs: Pitavastatin-incorporated PLGA NPs; UCNPs: platelet membranes were coated onto the surfaces of upconverting nanoparticles

**Table 5:** Preclinical operationalization of nanomedicine in ASCVD

NP strategy & category	Intervention timing	Treatment strategy (Preclinical)	Outcome assessment	Feasible clinical imaging modality	References
Anti-ROS-responsive (e.g. HA micelles, thioketal NPs)	Advanced/ vulnerable plaque: Targets established lesions to induce regression and reprogram the immune environment	Dosage: Repeated dosing. Every 3 days (HA-phenyl-boronate) or twice weekly (Thioketal-simvastatin) for 4—8 weeks	Readouts: • ↓ Atherosclerotic plaque area • ↓ ROS levels • ↓ proinflammatory cytokines (TNF-α, IL-1β) • ↓ necrotic core area	Non-invasive: CCTA, CMRA, or PCD-CT Invasive: OCT	[131, 132, 153–161]
Lipid-modulating (e.g. HDL-mimics NPs)	Early to advanced: To stabilize established lesions via cholesterol efflux	Dosage: Twice a week for 6 weeks	Readouts: • ↑ Cholesterol efflux • ↓ oxLDL uptake • ↓ Macrophage infiltration	Non-invasive: PET/CT or CMRA Invasive: OCT	[133, 153–161]
ECM & VSMC stabilization (e.g. Collagen IV or OPN-targeted NPs)	Acute injury/ regression stage: Targeted to exposed basement membranes or synthetic VSMCs to prevent restenosis	Dosage: Twice weekly (β-CD micelles) for 8 weeks or four times weekly for 2 weeks (OPN-peptide)	Readouts: • ↓ VSMC proliferation and migration • ↓ MMP activity (MMP-2/9) • Fibrous cap strengthening	Non-invasive: CCTA, advanced coronary CMRA or PCD-CT Invasive: OCT	[135, 146, 153–161]
Gene-editing (e.g. NP-mediated CRISPR/mRNA)	Early initiation: Permanent or long-acting modification of risk factors (e.g. PCSK9)	Dosage: Single systemic dose (one-off treatment)	Readouts: • ↓ PCSK9 level • ↓ LDL-C • ↓Atherosclerotic plaque area • ↓ Macrophage infiltration	Non-invasive: PET/CT or CMRA Invasive: OCT	[147, 148, 153–161]

HA: hyaluronic acid; ROS: ROS: reactive oxygen species; TNF-α: tumor necrosis factor alpha; IL-1β: Interleukin-1; CCTA: Coronary Computed Tomography Angiography; PET/CT: Positron Emission Tomography; PCD-CT: Photon-Counting Detector Computed Tomography; OCT: Optical Coherence Tomograph; HDL: high-density lipoprotein; oxLDL: oxidized LDL; LDL: low-density lipoprotein; ECM: extracellular matrix; VSMC: vascular smooth muscle cells; OPN: osteopontin; β‑CD: β-cyclodextrin; MMP: matrix metalloproteinase; PCSK9: proprotein convertase subilisin/kexin type 9

### Mechanosensing contributors in atherosclerosis

Mechanosensing is the ability of vascular cells to detect and convert physical forces into biochemical signals that lead to endothelial dysfunction and plaque progression in atherosclerosis. These processes are governed by the dynamic interplay of biomechanical changes, starting with disturbed hemodynamics. Nanomedicine can target mechanotransduction-related pathways to optimize diagnostic and therapeutic strategies for atherosclerosis in the future.

In diagnostic imaging using passive and active targeting strategies (Table 2), these approaches target specific microenvironments, receptors, and enzymes that are upregulated in response to biomechanical cues within atherosclerotic plaques. Passive targeting imaging leverages the ELVIS effect, where NPs accumulate in plaques due to increased vascular permeability [101]. Vulnerable plaques often exhibit an acidic environment (pH ~ 5.5) due to hypoxia and macrophage infiltration. Nanoprobes like IONP-HP are designed to respond to this acidic environment within lysosomes, specifically targeting the expression of CD14 and CD68 receptors [108]. Mechanically stressed cells generate elevated levels of •O^2−^ and ROS. RSPNs are employed in PAI to quantitatively measure •O^2−^ levels as a biomarker of plaque progression [110]. Nanoplatforms such as RBC/LFP@PMMP target lipid-rich inflammatory plaques by detecting oxLDL uptake, ROS overproduction, and MMP-9 activity [111]. Mechanical weakening of the fibrous cap is associated with elevated protease activity. Cathepsin B expression and phosphatidylserine (PS) receptor presentation on macrophage surfaces serve as key targets for enzyme-activatable fluorescent probes. [112].

Active targeting imaging employs ligand-functionalized NPs to target specific biomarkers associated with mechanical remodeling of the vessel wall. Thiolated glycol chitosan nanoprobes target mannose receptors on macrophages [116]. OPN, a phosphoprotein overexpressed in foam cells and macrophages in response to arterial stiffness, serves as a key target for UCNP probes to identify vulnerable plaques [117]. MPO activity, linked to plaque vulnerability and oxidative stress, is visualized using modified Fe₃O₄ NPs [120]. Physical forces and hypoxia further contribute to neovascularization, which can be exploited for diagnostic imaging. Diagnostic carriers target αvβ3 integrin activation and collagen type IV to detect early-stage plaques [123]. MMP-2 activity, an indicator of ECM degradation and fibrous cap thinning, is traced using AuNPs conjugated with MMP-2 antibodies for PAI [125].

Low and oscillatory wall shear stress (WSS) at arterial curvatures and bifurcations triggers several pathways, such as NF-kB and MAPK, and suppresses the protective KLF2/eNOS axis. In therapy, nanomedicine can provide access to this pathway through NP-mediated CRISPR formulations that knock out the mechanosensitive genes, such as thioredoxin domain containing 5 (TXNDC5), which prevents early atherogenesis [15, 162]. These mechanical processes significantly increase the recruitment of inflammatory immune cells that lead to drastically increased expression of *β*2-integrins on circulating hematopoietic cells, which are critical for monocyte adhesion to the activated arterial wall. To block this requirement, PEG-NPs can target overexpressed VCAM-1 while biomimetic macrophage-based NPs utilize integrin-VCAM interactions, which prevent monocyte adhesion [24, 163, 164].

Simultaneously, the progressive stiffening of the arterial wall, a hallmark of aging and lesion development resulting from elastin degradation and excessive, disorganized collagen content, serves as a critical mechanical stimulus. Vascular cells perceive this rigidity through transmembrane integrins, which link the ECM to the cytoskeleton and initiate intracellular signaling. Within this remodeled environment, fibronectin increases, providing an ECM base that increases mechanotransduction, facilitates pro-inflammatory signaling under mechanical load, and contributes further to arterial stiffness [165, 166]. The activation of these complexes is marked by increased FAK phosphorylation, a key downstream mediator that responds to the vessel wall rigidity. Moreover, these mechanical contributors drive a phenotypic switch in VSMCs. The arterial stiffness triggers downregulation of contractile markers such as ACTA2 and MYH11 in favour of a synthetic phenotype. This transition is identified by the overexpression of osteopontin, the marker of synthetic VSMCs associated with the mechanical environments that promote plaque instability. Mechanosensing transduction is further facilitated by mechanosensitive ion channels, most notably Piezo1 and the members of the transient receptor potential (TRP) family. Laminar flow induces Piezo1-mediated nitric oxide generation, whereas disturbed flow favors Piezo1-dependent inflammatory signaling [163]. Similarly, TRPV4 activation in macrophages promotes the uptake of oxLDL, accelerating the formation of foam cells and increasing lipid accumulation in plaques. The inflammatory signal is further defined by CD44-HA interactions, where the CD44 receptor on activated macrophages interacts with HA, and by elevated plaque cholesterol crystals that trigger the NLR family pyrin domain containing 3 (NLRP3) inflammasome. NPs can address these signals with micelles to significantly decrease oxLDL uptake [79, 162, 166]. As detailed in Table 3, nanomedicine platforms use these biological and mechanical indicators to achieve precise delivery. A variety of strategies target the ECM components, such as Type IV collagen and fibronectin, using hyaluronidase activity as triggers and/or MMP-2 activity as a target responsive drug release [124, 125]. Furthermore, cerium-based NPs, such as HA-CeO_2_, can scavenge ROS produced by mechanically stressed cells through SOD-mimetic activity, which leads to plaque area reduction [5, 31, 32]. By integrating these mechanosensing concepts, NPs can modulate pathways associated with atherosclerosis.

## Clinical studies of atherosclerosis therapy in nanomedicine

Nanomedicine platforms can serve as carriers to improve drug delivery and targeting efficiency. To overcome the limited bioavailability due to the low water solubility of fenofibrate, nanocrystal composite microparticles were used, with fenoglide becoming the first FDA-approved nanocrystal drug in 1993 [167]. Ultrasmall superparamagnetic iron oxide (USPIO) NPs were employed in the first MRI study of human atherosclerotic plaques [168]. Although nanomedicine offers promising treatment strategies for ASCVD in terms of precision medicine, there are currently only a few ongoing clinical trials. These NPs can be categorized on the basis of their biological targets or nanoplatforms. At present, lipid metabolism and macrophages within atherosclerotic plaques are among the most widely targeted mechanisms, whereas polymeric, lipid-based and liposomal NPs represent the most commonly used platforms in clinical trials (Table 6).

**Table 6:** Examples of NPs in ASCVD clinical trials

Start year	Study name	Study Design	Patient cohort	Number of patients	Nanomaterial/Cargo	Primary outcome	Study result	Reference; Clinical trial number
2007	NANOM-FIM	Multicenter, randomized, double-blind, observational	Patients with CAD and angiographic SYNTAX score ≤ 22	Total 180, 1:1:1 random (1) NPs in a bioengineered arterial patch (2) NPs with targeted microbubble and stem cell delivery (3) Stent implantation	Silica-gold	TAV by quantitative coronary angiography, intravascular ultrasound, MACE-free survival	Reduced TAV and risk of CV death. 5-year follow-up revealed high level safety, fewer MACE and less target lesion revascularization	[169, 170], NCT01270139
2011	CHI SQUARE	Phase II, multicenter, randomized, double-blind, placebo- controlled, ascending dose	Patients with ACS	Total 507 patients, 3:1 random (1) CER-001 (2) Placebo	An engineered pre-β HDL mimetic containing apo A-I and sphingomyelin (CER-001)	Change in total plaque volume assessed by IVUS	Didn’t reduce coronary atherosclerosis	[187], NCT01201837
2012	SILENCE	Phase I/II, randomized, double-blind, placebo- controlled	Patients with ASCVD	Total 30, 2:1 random (1) LNP-PLP (2) Placebo	Liposomes/Prednisolone (LNP- PLP)	Arterial wall permeability or inflammation assessed by FDG-PET/CT	LNP-prednisolone accumulated in the vessel wall, but had no anti-inflammatory effect	[176], NCT01601106
2015	CARAT	Phase II, multicenter, randomized, double-blind, placebo- controlled	Patients with ACS and baseline PAV greater than 30% in the proximal segment of an epicardial artery	Total 301 patients, 1:1 random (1) CER-001 (2) Placebo	An engineered pre-β HDL mimetic containing apo A-I and sphingomyelin (CER-001)	Nominal change in PAV in the target coronary artery by IVUS	Didn’t promote regression of coronary atherosclerosis in statin-treated patients with ACS and high plaque burden	[186], NCT02484378
2015	MILANO-PILOT	Phase I/II, multicenter, randomized, double-blind, placebo- controlled	Patients with ACS	Total 122 patients, 1:1 random (1) MDCO-216 (2) Placebo	HDL mimetic containing apo A-I (MDCO-216)	Change from baseline in PAV	Didn’t regress the coronary plaques in statin-treated patients with ACS	[197], NCT02678923
2016	TANGO	Phase III, multicenter, randomized, double-blind, placebo- controlled	Patients with genetically determined very low levels of HDL cholesterol and symptomatic or asymptomatic CVD	Total 30 patients, 2:1 random (1) CER-001 (2) Placebo	An engineered pre-β HDL mimetic containing apo A-I and sphingomyelin (CER-001)	Change in mean vessel wall area (MVWA) of the carotid artery	Didn’t change MVWA or arterial wall inflammation	[185], NCT02697136
2018	AEGIS-II	Phase III, multicenter, randomized, double-blind, placebo- controlled	Patients with AMI, multivessel coronary artery disease, and additional CV risk factors	18,226, 1:1 random (1) CSL-112 (2) Placebo	Human apo A-I with PC (HDL-like) (CSL-112)	Composite of MACCE from randomization through 90 days of follow-up	Didn’t reduce MACCE	[184], NCT03473223
2018	ORION	Phase III, randomized, double-blind, placebo- controlled	ORION-10: Patients with ASCVD ORION-11: Patients with CAD or ASCVD risk equivalent	ORION-10: 1561, 1:1 random ORION-11: 1617, 1:1 random	GalNac-Lipid NP (LNP)/ PCSK9 siRNA	Percent change in LDL-C	Approximately 50% reduction in LDL-C when given every 6 months	[177] NCT03399370, NCT03400800
2019	PAC-MAN	Phase II/III, randomized, double-blind, placebo- controlled	Patients with multi-vessel CAD	Total 38, 1:1 random (1) LDE-paclitaxel (2) LDE alone	Lipid-nanoemulsion (LDE)/ Paclitaxel	Low-attenuation plaque volume (LAPV) by coronary or aortic CTA	LDE-paclitaxel didn’t regress the coronary plaques	[175] NCT04148833,
2020	Raul et al	Phase II/III, randomized, double-blind, placebo- controlled	Patients with multi-vessel CAD	Total 40 (estimated), 1:1 random (1) MTX-LDE (2) LDE alone	LDE/methotrexate(MTX)	LAPV by coronary or aortic CTA	Ongoing with unknown status	NCT04616872
2022	Heart-1	Phage I, single-ascending dose, non- randomized	HeFH, ASCVD, and uncontrolled hypercholesterolemia patients	44 patients (estimated)	Lipid NP (LDL-like)/ PCSK9 CRISPR base editing (VERVE-101)	Incidence of TEAEs, SAEs and AESIs	Stopped due to safety concerns	[179], NCT05398029
2024	Heart-2	Phage I, single-ascending dose, non- randomized	HeFH or premature CAD patients	36 patients (estimated)	GalNac-Lipid NP (LDL-like)/PCSK9 CRISPR base editing (VERVE-102)	Incidence of TEAEs, SAEs and AESIs	Ongoing Preliminary data of first 14 patients showed no treatment-related SAEs in VERVE-102 treatment groups and 59% reduction of mean LDL-C from baseline if receiving a dose greater than 50 mg total RNA	NCT06164730

ACS: acute coronary syndrome; AESIs: adverse events of special interest; ASCVD: atherosclerotic cardiovascular disease; CAD: coronary artery disease; FDG-PET/CT: ^18^fluorodeoxyglucose positron emission tomography/computed tomography; HDL: high-density lipoprotein; HeFH: heterozygous familial hypercholesterolemia; IVUS: intravascular ultrasound; LAPV: Low-attenuation plaque volume; LDL-C: low-density lipoprotein cholesterol; MACCE: major adverse cerebrocardiovascular events, composite of myocardial infarction, stroke, or death from CV causes; MACE: Major Adverse Cardiovascular Events, composite of cardiac death, ST-elevation myocardial infarction (STEMI), non-STEMI and target lesion revascularization to the treated site; MVWA: mean vessel wall area; PAV: percent atheroma volume; SAEs: serious adverse events; TAV: total atheroma volume; TEAEs: treatment-emergent adverse events

### Inorganic

Photoactivatable photothermal agents are candidates for inducing macrophage apoptosis. In the first-in-human NANOM-FIM trial, compared with standard stent treatment, cellular hyperthermia caused by silica-gold-based NPs delivered via a bioengineered on-artery patch led to a reduction in atherosclerotic plaque and CV mortality [169, 170] (NCT01270139). This strategy, which uses various materials, has been investigated in multiple preclinical studies, and its advancement toward future clinical application is anticipated.

### Organic

#### Polymeric nanoparticles

Polymeric NPs have high stability and are resistant to phagocytosis by the reticuloendothelial system, thereby achieving prolonged blood circulation. Among these, HA-based and PLGA-based NPs are the most extensively studied for antiatherosclerotic applications. PLGA is renowned for its biocompatibility and controllable degradation rates. It has been approved by the US FDA for medical and pharmaceutical applications [171]. Currently, PLGA has been utilized in drug delivery systems in clinical trials [172]; however, clinical trials specifically targeting atherosclerosis with PLGA remain limited. Pitavastatin-incorporated PLGA NPs (NK-104-NPs) were formulated via an emulsion solvent diffusion method with intramuscular injections, which demonstrated good safety and tolerability in patients with chronic limb-threatening ischemia [173]. These authors reported that the blood concentration of pitavastatin increased in a dose-dependent manner. In addition, the PLGA polymer, a biodegradable polymer, has been widely applied in interventional stents to reduce possible stent-related side effects associated with biodurable polymers, such as in the SYNERGY™ stent system.

#### Lipid-based NPs

Lipid-based NPs, which are predominantly composed of lipids such as cholesterol and phospholipids, can easily cross the cell membrane, thereby significantly enhancing their cellular uptake. Therefore, they have been widely used as drug delivery carriers to target inflammation or lipid metabolism in ASCVD.

The Canakinumab Anti-Inflammatory Thrombosis Outcomes Study (CANTOS) reported that treatment with canakinumab, a monoclonal antibody against IL-1β, led to a reduction in nonfatal myocardial infarction, nonfatal stroke, and CV death in high-risk patients, which was independent of the effect of lipid-lowering agents [9]. In addition, the Colchicine Cardiovascular Outcomes Trial (COLCOT) and the Low-Dose Colchicine (LoDoCo) 2 trial indicated that colchicine, which suppresses inflammation by preventing cytoskeletal microtubule formation and inhibiting the nucleotide-binding oligomerization domain, leucine-rich repeat, and pyrin domain containing 3 (NLRP3) inflammasome, significantly lowers the risk of ischemic CV events in patients with recent MI and chronic coronary disease [7, 8]. On the other hand, the Cardiovascular Inflammation Reduction Trial indicated that low-dose methotrexate, an immune suppressant, did not decrease CV events in patients with stable ASCVD, as the levels of IL-1β, IL-6, and C-reactive protein did not change [174]. In addition, the CANTOS, COLCOT and LoDoCo trials revealed a higher incidence of infection, particularly pneumonia, in the treatment group. All of these studies demonstrated the potential of anti-inflammatory therapy for ASCVD; however, precise targeting to minimize systemic side effects remains a critical consideration. To achieve improved targeting efficiency and reduce adverse events, lipid-based NPs encapsulating anti-inflammatory drugs were further studied.

A lipid-nanoemulsion coating (LDE) that mimics the lipid composition of LDL has been developed, enabling uptake by LDL receptor-expressing cells such as inflammatory plaque macrophages. Recent phase II/III clinical trials have evaluated paclitaxel-loaded and methotrexate-loaded LDE formulations for their safety and efficacy in patients with stable coronary artery disease (CAD). Administration of LDE-paclitaxel every 3 weeks for 18 weeks was well tolerated but did not induce regression of the coronary plaques, as assessed by coronary computed tomographic angiography, in patients with multivessel CAD [175] (NCT04148833). A phase II/III randomized double-blind trial is still ongoing to assess the potential effect of methotrexate-loaded LDE on plaque regression in CAD patients (NCT04616872). Liposomal NPs with encapsulated prednisolone phosphate (LNP-PLP) were successfully taken up by plaque macrophages in patients with atherosclerotic diseases; however, they did not reduce arterial wall permeability or inflammation [176] (NCT01601106).

A number of promising new LNPs that modulate lipid metabolism and lower LDL-C have demonstrated outstanding efficacy. To increase liver uptake and specificity, N-acetyl galactosamine (GalNAc)-conjugated LNP encapsulation has been employed [177]. Recently, VERVE-101, a therapeutic LNP formulation using base-editing CRISPR/Cas9 technology, has provided excellent and durable inhibition of PCSK9, leading to long-acting reductions in LDL-C levels [147, 178]. A phase I clinical trial (NCT05398029) was initiated to evaluate the safety of VERVE-101 in patients with heterozygous familial hypercholesterolemia (HeFH), ASCVD, and uncontrolled hypercholesterolemia [179]. However, the trial was subsequently halted due to safety concerns, specifically grade 3 elevations in transaminases and grade 3 thrombocytopenia occurring within four days of administration. The safety of VERVE-102, which utilizes a GalNAc-LNP liver delivery system, was also investigated in a phase I trial enrolling patients with HeFH or premature CAD (NCT06164730). ANGLTL3, an endogenous inhibitor of lipoprotein lipase, is another therapeutic target for ASCVD. A GalNAc-LNP that targets ANGPTL3 via either CRISPR/Cas9 editing or siRNA has demonstrated a sustained reduction in ANGPTL3 levels in nonhuman primates [180, 181]. However, clinical trials investigating ANGPTL3-targeting treatments in humans have not yet been conducted.

Reverse cholesterol transport is the mechanism by which excess cholesterol is removed from peripheral tissues and transported back to the liver. Apolipoprotein A1 is the primary protein that mediates cholesterol efflux, the initial step of reverse cholesterol transport. CSL-112 is a human plasma-derived apolipoprotein A1 that is formulated with phosphatidylcholine to form disc-shaped NPs. These HDL-like NPs are more potent than native HDL in enhancing cholesterol efflux from macrophages, particularly through the efflux mediated by ATP-binding cassette transporter A1 [182, 183]. However, it failed to reduce major cerebral cardiovascular events, including myocardial infarction, stroke, or death from cardiovascular causes, in very high-risk patients (i.e., those with acute MI, multivessel CAD, and additional CV risk factors) [184] (NCT03473223). CER-001, an engineered preβ high-density lipoprotein (HDL) mimetic containing apolipoprotein A-I and sphingomyelin, was also tested in high-risk patients. However, it failed to reduce carotid vessel wall dimensions or arterial wall inflammation in patients with genetically determined very low HDL-C levels [185] (NCT02697136) or coronary atherosclerosis in ACS patients [186, 187] (NCT02484378, NCT01201837). These findings highlight that HDL-enhancing therapies for atherosclerosis might be more complex than previously anticipated and need further investigation.

## Challenges and future directions for ASCVD treatment via nanomedicine

Mainstream research in nanomedicine focuses on the design of nanocarriers to improve drug delivery, enhance target efficiency, and thereby optimize treatment outcomes. Despite rapid advances in nanotechnology and promising results from preclinical studies, several challenges remain in translating nanomedicine into clinical practice for ASCVD. These include the need for precise targeting of atherosclerotic plaques, the risk of off-target effects, potential NP-specific toxicities, and limitations in large-scale manufacturing due to batch-to-batch variability.

The biosafety, biodistribution, and clearance of NPs depend on several factors, including particle size, polarity, circulation dynamics, pharmacokinetics, and receptor affinity or binding properties [188]. The clearance of these particles is heavily dependent on their physicochemical properties. Most NPs are cleared through renal and hepatic pathways. For instance, HA-phenyl-boronate micelles tend to accumulate in the filtering organs like liver and kidney, while cerium-based nanozymes and macrophage-specific HDL-mimics show high affinity to the liver and spleen (Table 3). The appropriate biodistribution of NPs is critical for clinical applications, as it determines the targeted efficacy as well as the risk of acute or chronic side effects. Importantly, these applications become particularly relevant in repeated dosing, where NPs accumulate in off-target cells, tissues, or organs, and altered pharmacokinetics may influence long-term safety and therapeutic effectiveness. Nanotechnology can enhance the absorption and distribution of drugs by facilitating the delivery of one or more therapeutic drugs, thereby improving overall efficacy. In this context, precise targeting remains the main advantage of NPs over small-molecule drugs; however, several challenges persist regarding targeting efficiency and potential adverse effects.

Targeted strategies are informed by the pathophysiology of plaque formation, in which LDL-C accumulation, impaired cholesterol efferocytosis, and local inflammation are central features. Current designs leverage HDL-like or LDL-like NPs to modulate inflammation and lipid composition within atherosclerotic plaques. However, lipid-based NPs themselves may paradoxically exacerbate arterial inflammation in the lipid-rich milieu [189], potentially offsetting the anti-inflammatory effects of the encapsulated drugs. In addition, NPs might induce endothelial leakage and inflammation, promote the phenotypic switching and proliferation of VSMCs, and activate immune responses [190]. Therefore, the physicochemical properties of NPs, including their size, surface charge, protein corona, and cargo composition, require careful optimization to minimize immune interactions and other side effects in future clinical applications. Recently, nucleic acid-based nanotherapeutics incorporating functional ligands, such as inclisiran, have demonstrated improved efficacy and reduced toxicity. Similar to the recognition of GalNAc by hepatocytes, NPs conjugated with the peptide VHPKQHR, which targets VCAM-1, selectively bind inflamed endothelial cells and deliver genes upregulated under pathological conditions [191]. This ligand-based targeting strategy might represent a promising direction for future ASCVD therapies.

Although the safety and efficacy of NPs have been demonstrated in rodent studies, preclinical studies cannot reliably predict their efficacy in humans. For example, genetically manipulated models fail to reflect the polygenic nature of human diseases and the substantial influence of environmental factors. In addition, disease progression and pathological features in animal models differ significantly from those observed in humans. Therefore, the identification of novel therapeutic targets in mice should be conducted in parallel with investigations using human biobanks to ensure clinical relevance. For example, phospholipid phosphatase 3 (PLPP3), an integral membrane protein that inactivates lysophosphatidic acid, has been implicated in CAD through genome-wide association studies. Its single-nucleotide polymorphism has been shown to influence critical endothelial mechanotransduction mechanisms [192]. Further research demonstrated that the delivery of PLPP3 mRNA by VCAM-1-treated LNPs effectively attenuated arterial inflammation, slowed atherosclerosis progression, and promoted the regression of advanced plaques (preprint in bioRxiv). These findings suggest that gene targets with human disease relevance, combined with precise targeting strategies delivered by biocompatible NPs, represent a promising direction for nanomedicine in ASCVD. Clinical trials are essential for translating laboratory findings into clinical applications. Despite promising laboratory formulations, limited information is available about the technologies required for the large-scale production of nanomedicines. Moreover, industrial-scale NP production must comply with good manufacturing practice (GMP) standards to ensure batch-to-batch reproducibility. NP manufacturing techniques are typically classified into top-down or bottom-up approaches, and various methods have been explored to optimize large-scale NP production [193–195]. The manufacturing, characterization and process control employed in the production of inclisiran serve as valuable examples for future NP production. In addition, novel platforms have been developed to enhance the self-assembly of carriers, such as membrane-anchored lipoproteins incorporated into siRNA-loaded LNPs that interact with the Fc domain of antibodies [196]. Even though further improvements are needed, the scale-up manufacturing of NPs is advancing and remains a key area for future translational success.

## Conclusions

Given the complexity of atherosclerosis pathogenesis and the high residual risk in ASCVD patients despite current guideline-directed treatment, precision and plaque-targeted therapies for ASCVD remain crucial yet challenging. Nanomedicine, which integrates biocompatibility, tissue targeting, cellular mechanisms, and drug-loading capacity, could offer significant advantages for mechanism-driven diagnosis and treatment with high cell specificity. Although preclinical trials have yielded promising results, clinical trials are essential to translate these laboratory findings into practical applications. It also shows certain potentials of nanomedicine-based diagnostic imaging/theranostics to integrate with guideline-directed therapy and revascularization in different stages (Fig. 3). Nonetheless, several limitations and challenges still hinder the clinical translation of nanomedicine for ASCVD. Future efforts should focus on providing stronger clinical evidence and overcoming these anticipated obstacles in this field.

## Data Availability

No datasets were generated or analysed during the current study.

## Abbreviations

AAV: Adeno-associated virus
AM: Amphiphilic macromolecule
ApoE: Apolipoprotein E
ASCVD: Atherosclerotic cardiovascular diseases
AuNPs: Gold-based NPs
CAD: Coronary artery disease
COLCOT: Colchicine cardiovascular outcomes trial
cRGD: Cyclic-arginine-glycine-aspartic
CRISPR: Clustered regularly interspaced short palindromic repeats
CT: Computed tomography
CV: Cardiovascular
d-HDL: Discoidal HDL
ECM: Extracellular matrix
eNOS: Endothelial nitric oxide synthase
FAK: Focal adhesion kinase
FC: Ferrocene
FDA: Food and drug administration
GalNAc: N-acetyl galactosamine
GMP: Good manufacturing practice
GOQD: Graphene oxide quantum dot
H&E: Hematoxylin and eosin
H₂O₂: Hydrogen peroxide
HA: Hyaluronic acid
HDL: High-density lipoprotein
ICAM-1: Intercellular adhesion molecule-1
ICG2: Indocyanine green 2
IL-10: Interleukin-10
IL-1β: Interleukin-1 beta
IONP-HP: Iron oxide nanoprobe
IONPs: Iron oxide-based NPs
KLF2: Krüppel-like factor
LCAT: Lecithin-cholesterol acyltransferase
LDE: Lipid-nanoemulsion coating
LDL-C: Low-density lipoprotein cholesterol
LNPs: Lipid-based nanoparticles
MMPs: Metalloproteinases
MMR: Macrophage mannose receptor
MRI: Magnetic resonance imaging
MSNs: Mesoporous silica-based NPs
NF-κB: Nuclear factor kappa B
NIRF: Near-infrared fluorescence
NO: Nitric oxide
NPs: Nanoparticles
OPN: Osteopontin
oxLDLs: Oxidized low-density lipoproteins
PAAO: Polyacrylate-n-octyl amine
PAI: Photoacoustic imaging
PEG: Polyethylene glycol
PET: Positron emission tomography
PLGA: Poly(lactic-co-glycolic acid
PLPP3: Phospholipid phosphatase 3
PPAR-γ: Peroxisome proliferator-activated receptor gamma
Ptyr: Poly(tyrosine-ethyl oxalyl
r-HDL: Recombinant HDL
RBC: Red blood cell
ROS: Reactive oxygen species
RSPNs: Ratiometric semiconducting polymeric nanoparticles
s-HDL: Spherical HDL
SARS-CoV-2: Severe acute respiratory syndrome coronavirus 2
scFv: Single-chain variable fragment
SHP-1: Src homology region 2 domain-containing phosphatase-1
SOD: Superoxide dismutase
TLE: Thin-layer evaporation/extrusion
TNF-α: Tumor necrosis factor alpha
TRAF6: Tumor necrosis factor receptor associated factor 6
TRP: Transient receptor potential
UCNP: Upconversion nanoparticle
USPIO: Ultrasmall superparamagnetic iron oxide
VCAM-1: Vascular cell–adhesion molecule-1
VEGF: Vascular endothelial growth factor
VSMCs: Vascular smooth muscle cells
WSS: Wall shear stress
WST-8: Water-soluble tetrazolium 8
ZIF-8: Zeolitic imidazolate framework
